# Enhancing drought tolerance in *Malva parviflora* plants through metabolic and genetic modulation using *Beauveria bassiana* inoculation

**DOI:** 10.1186/s12870-024-05340-w

**Published:** 2024-07-11

**Authors:** Reda E. Abdelhameed, Elham R. S. Soliman, Hanan Gahin, Rabab A. Metwally

**Affiliations:** 1 Botany and Microbiology Department, Faculty of Science, Zagazig University, Zagazig, 44519 Egypt; 2 Cytogenetics and Molecular Genetics Unit, Botany and Microbiology Department, Faculty of Science, Helwan University, Helwan, 11795 Egypt

**Keywords:** Ascorbic acid, Hormones, Lipoxygenase, Secondary metabolism, Genetic variation, Genome stability

## Abstract

**Background:**

Enhancing crops’ drought resilience is necessary to maintain productivity levels. Plants interact synergistically with microorganisms like *Beauveria bassiana* to improve drought tolerance. Therefore, the current study investigates the effects of biopriming with *B. bassiana* on drought tolerance in *Malva parviflora* plants grown under regular irrigation (90% water holding capacity (WHC)), mild (60% WHC), and severe drought stress (30% WHC).

**Results:**

The results showed that drought stress reduced the growth and physiological attributes of *M. parviflora*. However, those bioprimed with *B. bassiana* showed higher drought tolerance and enhanced growth, physiological, and biochemical parameters: drought stress enriched malondialdehyde and H_2_O_2_ contents. Conversely, exposure to *B. bassiana* reduced stress markers and significantly increased proline and ascorbic acid content under severe drought stress; it enhanced gibberellic acid and reduced ethylene. Bioprimed *M. parviflora*, under drought conditions, improved antioxidant enzymatic activity and the plant’s nutritional status. Besides, ten Inter-Simple Sequence Repeat primers detected a 25% genetic variation between treatments. Genomic DNA template stability (GTS) decreased slightly and was more noticeable in response to drought stress; however, for drought-stressed plants, biopriming with *B. bassiana* retained the GTS.

**Conclusion:**

Under drought conditions, biopriming with *B. bassiana* enhanced *Malva*’s growth and nutritional value. This could attenuate photosynthetic alterations, up-regulate secondary metabolites, activate the antioxidant system, and maintain genome integrity.

**Supplementary Information:**

The online version contains supplementary material available at 10.1186/s12870-024-05340-w.

## Highlights

Drought exposure altered the physiological and biochemical functioning of *M. parviflora* plants and results in impaired growth, water status and reduced pigment fractions.

*Beauveria bassiana* bio-priming improves the physiological functioning of *M. parviflora* plants and drought stress resilience.

Drought stress augmented the oxidative stress in plants by over-increasing malondialdehyde and H_2_O_2_ contents. However, *B. bassiana* decreased their levels in bioprimed *M. parviflora* plants.

*B. bassiana* improved drought tolerance through upregulating the production of secondary metabolites, antioxidants, the modulation of phytohormones, maintaining genome template stability and converting unavailable nutrients into available forms and increasing their absorption by *M. parviflora* plants.

**Supplementary Information:**

The online version contains supplementary material available at 10.1186/s12870-024-05340-w.

## Background

Climate change is one of the most serious global threats to agriculture; drought, one of climate change’s results, poses a global threat to food security because it impedes crop growth and productivity [1, 2]. Drought has caused significant famines in the past and is still the primary threat to future food security [3], with the considerable hazard that by 2050, 50% of cultivable land will face severe water shortages and droughts. Water stress has long been recognized as a significant factor influencing plant growth at various stages of development and crop production [4].

Drought stress induces several physiological and biochemical changes. It directly affects the photosynthetic apparatus, particularly disrupting photosynthetic process components, i.e., thylakoid electron transport, the stomatal conductance of CO_2_ supply and carbon reduction cycle, lipid peroxidation, and cellular water balance disturbance. Additionally, decreased cell division, cell elongation, and excessive oxidative damage can all negatively affect biomass [2]. Water stress also causes an imbalance between the generation of reactive oxygen species (ROS) and antioxidative defense mechanisms, resulting in elevated ROS accumulation, which leads to oxidative stress on proteins, lipids, and DNA fragments [5].

To cope with drought stress, plants initiate defense strategies categorized as morphological, physio-biochemical, and genetic mechanisms. The main attributes of morphological mechanisms include drought escape, drought avoidance, and phonological flexibility, which can be achieved through early growth vigor, reduced leaf area, and a vigorous root system. The most significant physio-biochemical mechanisms underlying drought tolerance involve osmotic adjustment, osmoprotection, antioxidation, and scavenging defense systems [6, 7]. Genetically, plants can adapt to drought stress by modulating the expression of early responsive genes involved in plant tolerance, such as genes encoding ROS and osmolytes [8]; otherwise, they can generate DNA mutations that improve plant performance [9].

Endophytes are microbes that reside in healthy plant tissues without causing any damage. They penetrate the plant *via* natural openings, such as stomatal openings [10, 11]. These endophytes promote plant development and disease resistance in numerous ways [12]. For instance, they produce and release secondary metabolites and biochemicals which protect plants from pathogens [13]. Endophytes that promote plant development are known for their role as drought-tolerance enhancers, demonstrating various strategies for dealing with the drought’s impacts on soil and plants via adjusting the molecular, physiological, and biochemical changes that occur in the plant. These microbes affect plants through three different methods: (1) they change phytohormonal activity, (2) deposit osmolytes that aid in plant resistance to drought, and (3) protect plants with antioxidants [14, 15].

The fungal genus *Beauveria* (Hypocreales: Cordycipitaceae) is considered both entomopathogens and endophytic plant symbionts. This fungus may colonize various plant species, protect them from pests, and boost their development and nutritional value [16]. Common beans and lettuce inoculated with *B. bassiana* grow faster and have better physiological and antioxidant activity [17, 18]. In this regard, Ferus et al. observed that *B. bassiana* enhanced drought tolerance in red oak seedlings [19]. Moreover, many studies have found that endophytes serve as bio-stimulants to promote macro- and micronutrient uptake and translocation that ultimately modify phytohormones to improve plant growth [18, 20]. Fungal endophytes can solubilize phosphate, generate phytohormones such as indole acetic acid (IAA), gibberellic acid (GA), and siderophore [21, 22], and encourage plants to produce antioxidants, crucial for ROS scavenging [11, 20].

*Malva parviflora* L., also known as cheeseweed or little mallow, is a widely common species in the genus *Malva* and family Malvaceae; it is an annual or biennial herb native to Europe, Asia, and Africa, cultivated for both food and medicinal purposes [23, 24]. Given its nutritional value and palatability, it can be developed as a specialty leafy vegetable crop, but additional research and breeding efforts would be required to support commercial cultivation. Although many studies investigated the effects of water stress and fungal endophytes on plant growth and secondary metabolites [11, 25–27], only a few studies focused on using *M. parviflora* under other abiotic stresses [28–30]. To enhance the growth of *M. parviflora* under drought stress, the current study investigated the use of the endophytic *B. bassiana* as a priming agent by addressing the impact of *B. bassiana* fungal colonization on *M. parviflora* growth, as well as its physiological (i.e., chlorophyll contents and water status) and biochemical responses (i.e., osmolytes, stress biomarkers, and enzymatic and non-enzymatic antioxidants) to drought stress. In addition, the study examined the genetic alterations in *M. parviflora* in response to drought conditions, both with and without fungal inoculation with *B. bassiana*.

## Results and discussion

### Molecular identification of *B. bassiana*

**Fig. 1:**
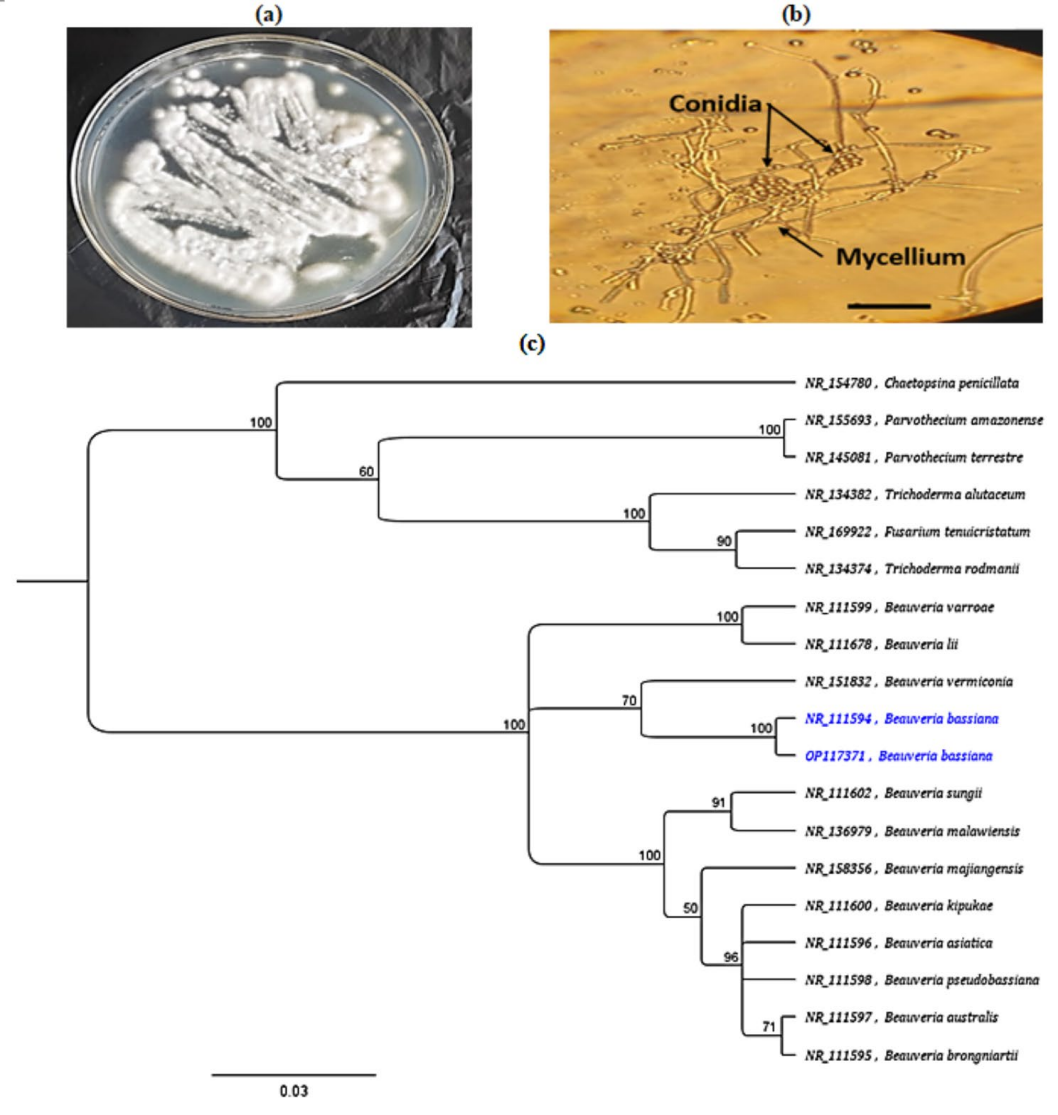
(**a**) *B. bassiana* grown in Potato dextrose agar (PDA), (**b**) Photograph of *B. bassiana* under a compound microscope and (**c**) The phylogenetic tree of the ITS rDNA region for *B. bassiana* RA1 (OP117371) isolate (blue font) and the others presented on GenBank based on the ITS rDNA sequences

The morphological identity of *B. bassiana* RA1 (Fig. 1a, b) was validated by molecular analysis of the ITS rDNA sequence (18–28 S rRNA encoding gene), flanking ITS1 (5.8 S rRNA), and ITS2, employing ITS1/ITS4 primers. The obtained ITS sequence was entered into NCBI GenBank with the accession number OP117371. The sequence data were aligned using the BLAST program to assess their similarity to related strains. Based on ITS sequences, the phylogenetic tree displayed in Fig. 1c was created using the neighbor-joining method with 1000 bootstrap repetitions. The closets homologous to the sequences were selected, and multiple sequence alignments were carried out using the ClustalW program in the MEGA7 version 7 software [31].

### Colonization of *M. Parviflora* roots

Typical *B. bassiana* colonies were isolated from *M. parviflora* roots and confirmed by PDA culturing, observing the hyphal, and conidial structures (see Fig. 1b). All *B. bassiana*-treated *M. parviflora* showed mycelial outgrowth on their sections, representing 100% fungal colonization. No fungal outgrowth occurred in the no-fungus *M. parviflora* (i.e., the control samples).

### Effect of *B. bassiana* on the *M. parviflora*’ growth traits under drought stress

**Fig. 2:**
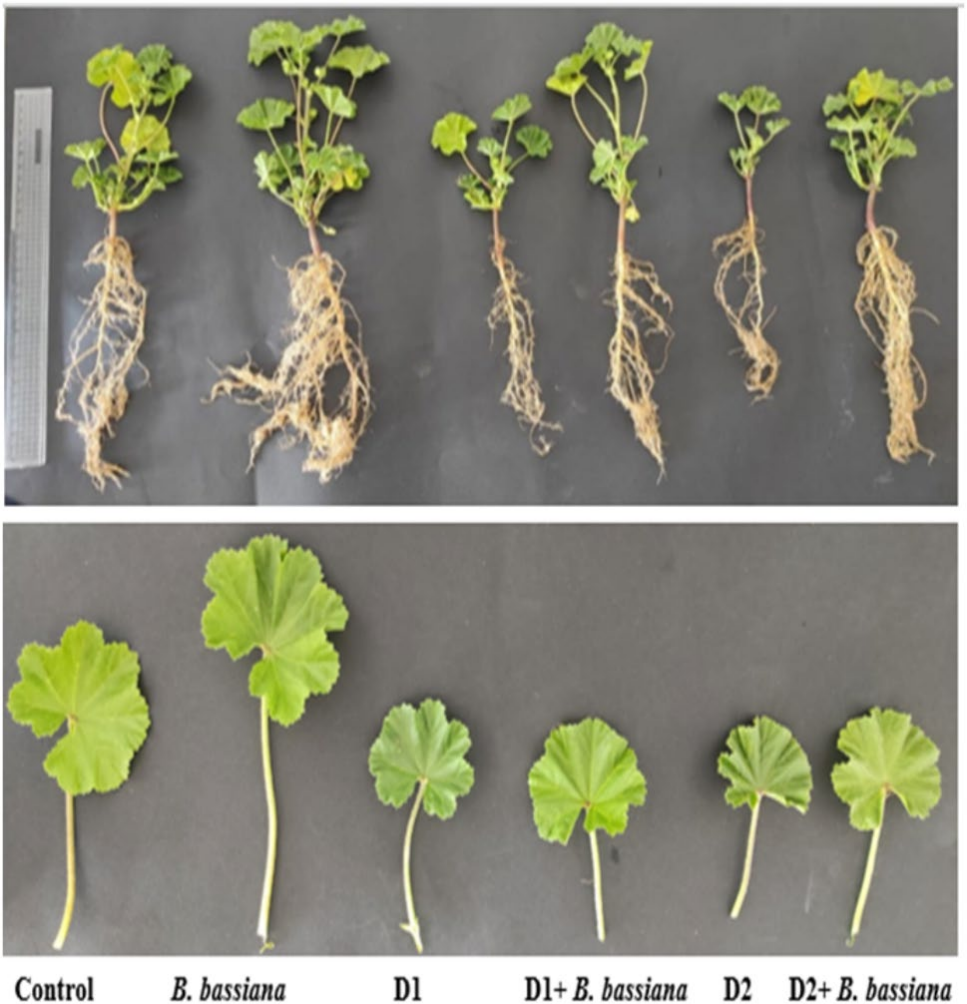
Photograph of *M. parviflora* plants uninoculated and inoculated with *B. bassiana* under drought stress conditions. **Control**: normal irrigation (90% WHC “water holding capacity”), **D1**: mild drought stress (60% WHC), and **D2**: severe drought stress (30% WHC)

Earlier reports showed that seed biopriming with a biocontrol agent improved crops [32, 33] due to better colonization adaptability and suitability under biotic and abiotic stress conditions. The following growth parameters including shoot, root, and petiole length; leaf number; fresh weight (FW) of leaves; their dry weight (DW); and leaf area were evaluated to assess the effects of *B. bassiana* fungal inoculation on *M. parviflora*’s growth under severe drought stress (30% WHC), moderate drought stress (60% WHC), and controlled conditions (90% WHC). Table 1 shows that these growth parameters were reduced in drought-stressed *M. parviflora* compared to controls. Our results agree with Abobatta’s [34] that drought stress suppresses cell expansion and growth due to low turgor pressure. Consequently, at a morphological level, plants decrease leaf number and area to adapt to drought conditions. However, biopriming *M. parviflora* with *B. bassiana* positively affected these growth parameters (Fig. 2). Under severe drought stress, shoot height, root length, leaf number, shoot, and root FW increased by 47.5, 17.8, 60.0, 39.0, and 62.3% in *B. bassiana-*treated plants as compared to non-treated ones, respectively (see Table 1).

**Table 1:** Mean of growth parameters of *M. parviflora* plants (control) and plants bioprimed with *B. bassiana* under drought stress conditions

Parameters Treatments	Shoot length (cm)	Root length (cm)	Shoot FW (g)	Root FW (g)	Shoot DW (g)	Root DW (g)	Leaves number	Petiole length (cm)	Leaf FW (g)	Leaf DW (g)	Leaf area (cm^2^)
**Control**	11.9 ± 0.315b	21 ± 0.555b	9.47 ± 0.251b	6.86 ± 0.181b	2.93 ± 0.077b	1.17 ± 0.031d	11 ± 0.291b	6.2 ± 0.164b	0.51 ± 0.013b	0.19 ± 0.005b	4.5 ± 0.119b
***B. bassiana***	14.5 ± 0.384a	24.5 ± 0.648a	12.6 ± 0.333a	9.15 ± 0.242a	3.42 ± 0.09a	2.410 ± 0.064a	15 ± 0.396a	7.7 ± 0.204a	0.81 ± 0.021a	0.25 ± 0.0066a	5.1 ± 0.135a
**D1**	7.3 ± 0.193d	17 ± 0.449d	5.05 ± 0133d	4.83 ± 0.128c	1.76 ± 0.046d	0.83 ± 0.022e	7 ± 0.185e	5.7 ± 0.151c	0.4 ± 0.011 cd	0.13 ± 0.003d	3.8 ± 0.1005c
**D1 +** ***B. bassiana***	10.5 ± 0.277c	19 ± 0.503c	7.85 ± 0.207c	6.36 ± 0.168b	2.06 ± 0.054c	1.58 ± 0.042b	10 ± 0.265c	6.1 ± 0.161bc	0.44 ± 0.012c	0.16 ± 0.004c	4.4 ± 0.116b
**D2**	4 ± 0.106f	14 ± 0.371e	3.99 ± 0.105e	3.08 ± 0.081d	0.99 ± 0.026e	0.58 ± 0.015f	5 ± 0.132f	4 ± 0.106e	0.36 ± 0.009d	0.11 ± 0.0029e	2.9 ± 0.077d
**D2 +** ***B. bassiana***	5.9 ± 0.156e	16.5 ± 0.436d	5.55 ± 0.146d	5 ± 0.132c	1.79 ± 0.047d	1.45 ± 0.038c	8 ± 0.212d	4.5 ± 0.119d	0.39 ± 0.0103d	0.13 ± 0.0034d	3.7 ± 0.098c

Control: normal irrigation (90% WHC “water holding capacity”), D1: mild drought stress (60% WHC), and D2: severe drought stress (30% WHC). Data are the mean (± SE; *n* = 5). Means of the same column followed by the same letter are not significantly different (*p* < 0.05) according to One-Way ANOVA: Post Hoc Multiple Comparisons (Duncan)

Consistent with Afandhi et al.’s findings [17], *B. bassiana* inoculation facilitates the transmission of growth-promoting nutrients from the soil to the common bean plants. Gana et al. [1], Jaber [35], and Russo et al. [36] reported similar effects on wheat, corn, and onion plants, respectively. Endophytes’ beneficial effects include promoted plant growth, improved nutrient uptake, and increased tolerance to abiotic stress [37, 38]. The enhanced root development by *B. bassiana* (as shown in Fig. 2) may affect the plant’s survival rate, given that the plant can achieve higher stability and easily reach nutrients from the soil. Besides, endophytes directly or indirectly produce several bioactive compounds with various biological activities. These compounds are plant growth-promoting (PGP) agents and protectors that improve root and shoot systems by increasing root length, plant height, FW, and DW [39, 40].

### Water status of *M. parviflora* under drought stress and *B. bassiana* treatment

**Fig. 3:**
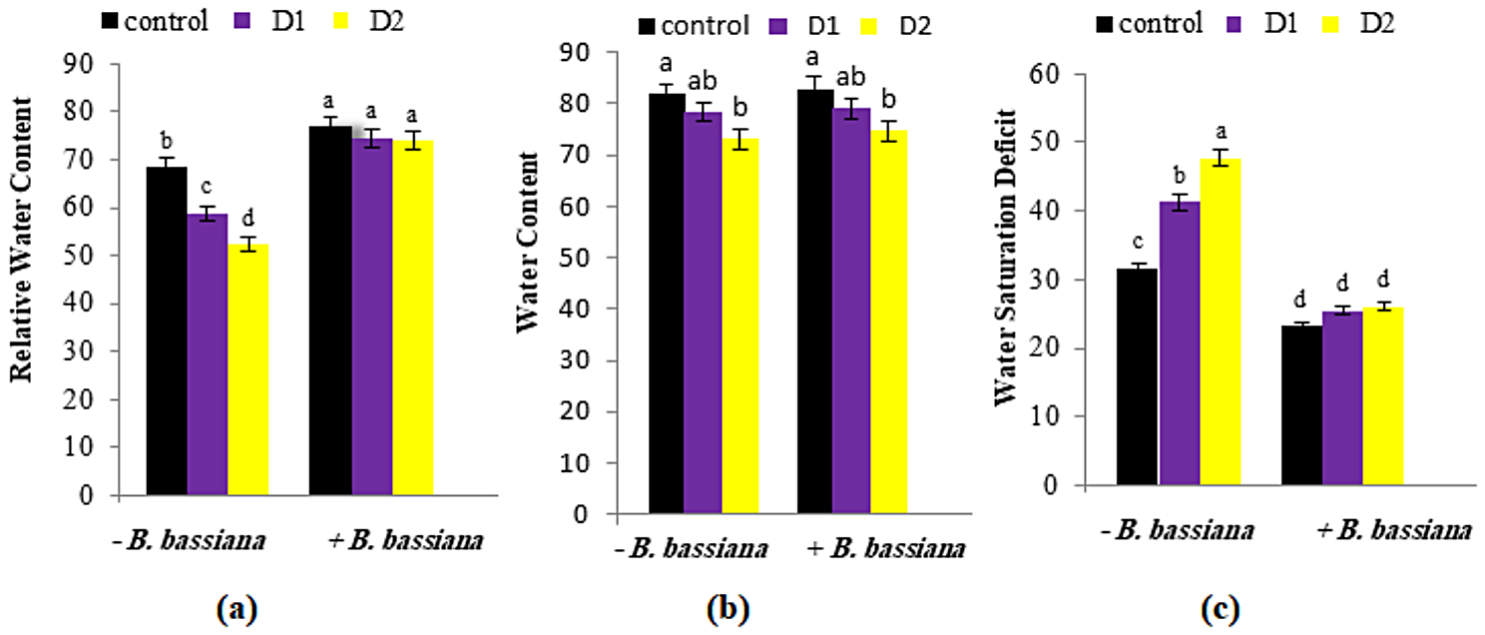
Effect of *B. bassiana* inoculation on the water status: (**a**) relative water content (RWC), (**b**) water content (WC) and (**c**) water saturation deficit (WSD) in *M. parviflora* L plants under drought stress. Data are the mean (*n* = 5). Error bars represent the standard error of the mean and different letters show significant differences at *p* < 0.05 according to One-Way ANOVA: Post Hoc Multiple Comparisons (Duncan)

The leaves’ water status is a crucial characteristic directly related to soil water content and an essential indicator of the leaf’s metabolic activity and survival. Figure 3 (a-c) showed that the water content (WC) and relative water content (RWC) were decreased in drought-stressed *M. parviflora* leaves compared to the control plant. However, the water saturation deficit (WSD) increased with drought stress. The reduction percent in WC and RWC in *M. parviflora* leaves significantly increased under severe drought conditions compared to moderate ones. Drought stress affects plant water status by decreasing water availability, leaf water potential, and transpiration rate and increasing leaf temperature due to the negative effect of drought on stomatal opening and closing processes [41].

As a result, *B. bassiana* treatment improved water status and increased WC and RWC in *M. parviflora* under drought stress. Besides, the plants that interacted with beneficial endophytes used less water, increased biomass, reduced leaf conductance, and slowed transpiration during drought stress [42, 43]. Hosseini et al. [44] reported that *Piriformospora indica* enhanced maize water status and physiological traits under combined drought and mechanical stresses by increasing root volume, leaf area, RWC, leaf water potential, and proline content. Moreover, Llorens and collaborators [45] detected a higher RWC, proline, abscisic acid, and jasmonic isoleucine compound in endophyte-treated plants than untreated ones.

Previous studies have confirmed the potential of *B. bassiana* to increase water use efficiency under stress conditions [46–48]. According to Ferus et al. [19], the leaf RWC of red oak seedlings decreased after drought stress application in non-*B. bassiana*-colonized seedlings; however, this decrease was remarkably diminished with *B. bassiana* application. Akter et al. [48] stated that the *B. bassiana*-primed rice plants showed improvement in shoot RWC, leading to enhanced growth under both salt stress and control conditions. The expected explanation for *B. bassiana*^’^s beneficial effect on enhancing *M. parviflora* water availability was only recognized through root growth improvement under mild and severe drought stress. As a result, *B. bassiana* can successfully assist *M. parviflora* in coping with drought stress.

### Influence of *B. bassiana* on photosynthetic pigments of drought-stressed plants

The levels of Chl a and b, carotenoids, and total pigments in *M. parviflora* leaves were adversely affected by drought stress and sharply dropped, especially under extreme drought circumstances (Table 2). Chl a, b, and carotenoid levels dropped under extreme drought conditions by 45.4, 23.4, and 34.4%, respectively, compared to those under control conditions. Moderate and severe drought stress reduced the total pigment levels by 23.9 and 37.8%, respectively, compared to the control levels. Related results were observed in drought-stressed *Moringa oleifera* and *Avena nuda* plants [11, 49]. Drought stress causes pigment degradation by upregulating chlorophyllase activity and downregulating other related enzymes in Chl production. According to Anjum et al. [50], under drought stress, the decrease in Chl content was a sign of photo-oxidation because drought causes stomatal closure, decreased CO_2_ entry, and impaired photosynthetic rate. It also leads to an imbalance in the light-harvesting complex I and II and changes the photochemistry in chloroplasts, producing excess ROS. Table 2 illustrates a significant difference between untreated and *B. bassiana*-treated plants; under both control and drought stress circumstances, *B. bassiana* inoculation significantly enhanced Chl a and Chl b levels in *M. parviflora* leaves due to the plants’ improved ability to absorb nutrients and water, both of which are necessary for producing pigments utilized in photosynthetic processes [51]. Additionally, *B. bassiana* inoculation might influence plant physiological functions and cell wall composition, promoting the production of proteins and enzymes involved in color manufacturing [52].

**Table 2:** Mean of plant pigment fractions (mg/g FW) of *M. parviflora* plants (control) and plants inoculated with *B. bassiana* under drought stress conditions

Parameters Treatments	Chl a	Chl b	Total Chl	Carotenoids	Total pigments	Chl a/ Chl b
**Control**	1.109 ± 0.029b	0.414 ± 0.011ab	1.523 ± 0.04b	0.706 ± 0.0187a	2.229 ± 0.059b	2.678 ± 0.071b
***B. bassiana***	1.271 ± 0.034a	0.421 ± 0.011a	1.692 ± 0.045a	0.737 ± 0.0195a	2.429 ± 0.064a	3.019 ± 0.079a
**D1**	0.784 ± 0.021d	0.397 ± 0.0105ab	1.181 ± 0.031d	0.515 ± 0.0136b	1.696 ± 0.045d	1.975 ± 0.052de
**D1 +** ***B. bassiana***	0.988 ± 0.026c	0.423 ± 0.0112a	1.411 ± 0.037c	0.545 ± 0.0144b	1.956 ± 0.052c	2.336 ± 0.062c
**D2**	0.605 ± 0.016e	0.317 ± 0.008c	0.922 ± 0.024e	0.463 ± 0.0122c	1.385 ± 0.037e	1.908 ± 0.05e
**D2 +** ***B. bassiana***	0.818 ± 0.022d	0.381 ± 0.01b	1.199 ± 0.032d	0.512 ± 0.0135b	1.711 ± 0.045d	2.1469 ± 0.056 cd

Data are the mean (± SE; *n* = 5). Means of the same column followed by the same letter are not significantly different (*p* < 0.05) according to One-Way ANOVA: Post Hoc Multiple Comparisons (Duncan)

### *B. Bassiana* adjusted the synthesis of compatible solutes in drought-stressed *M. parviflora*

**Fig. 4:**
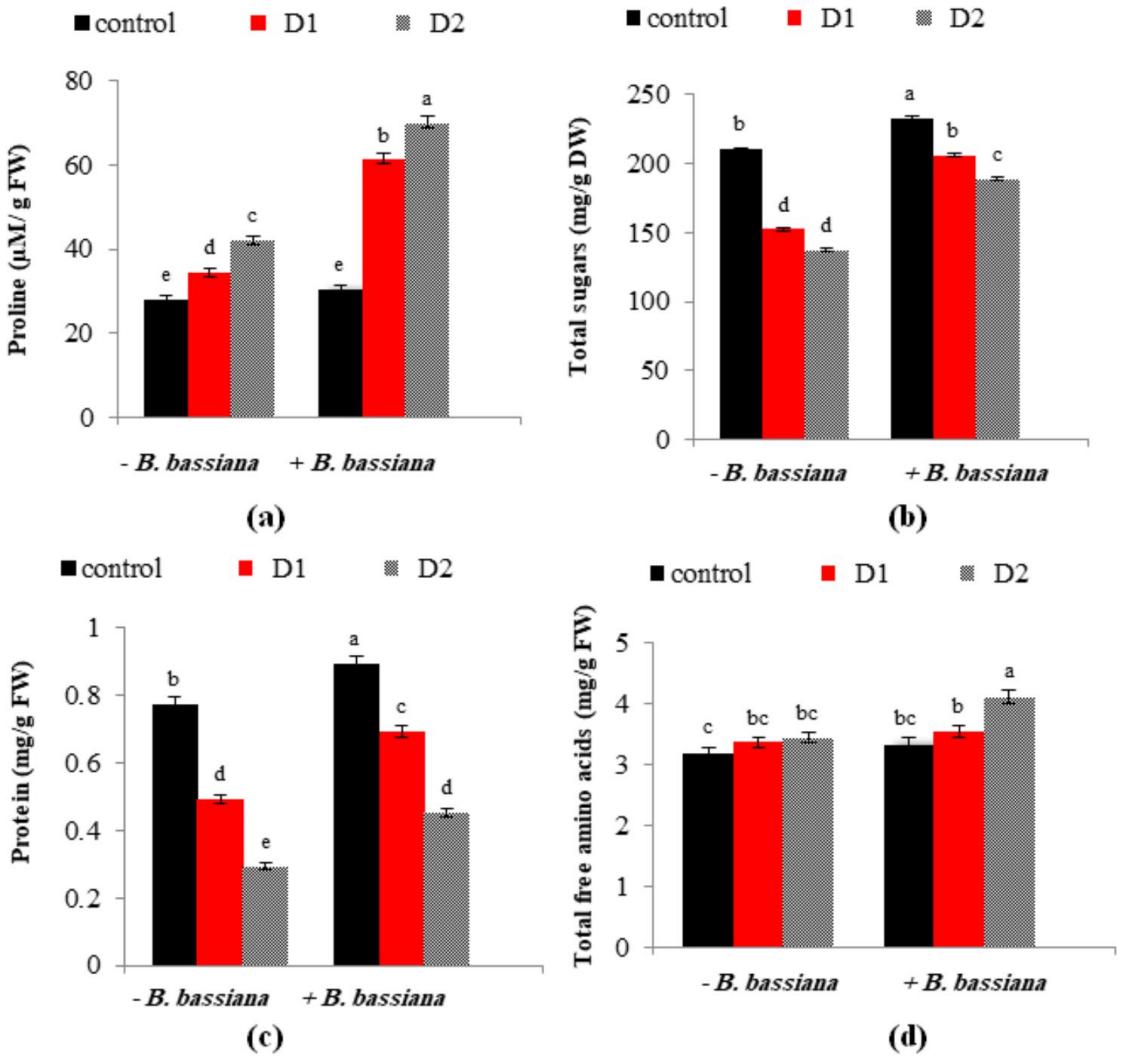
Effect of *B. bassiana* inoculation on the osmolytes content: (**a**) proline, (**b**) total soluble sugars, (**c**) total soluble protein and (**d**) total free amino acids in *M. parviflora* L plants under drought stress. Data are the mean (*n* = 5). Error bars represent the standard error of the mean and different letters show significant differences at *p* < 0.05 according to One-Way ANOVA: Post Hoc Multiple Comparisons (Duncan)

In the current study, cellular osmolytes were altered by the effects of drought and *B. bassiana* application, as shown in Fig. 4 and Table 3. Proline and amino acid levels in drought-stressed *M. parviflora* were elevated (Fig. 4a, d), demonstrating the physiological changes caused by drought aggravation. Compared to those grown under well-irrigated conditions, the proline content of *M. parviflora* leaves increased by 23.2 and 50.5% under moderate and severe drought circumstances. Conversely, total soluble protein and carbohydrates showed a different trend as drought stress decreased their contents in *M. parviflora* leaves (Fig. 4b, c). In accordance with our results, the increased accumulation of proline and amino acids constitutes a general adaptive mechanism developed by plants to counteract stress conditions and safeguard the structural integrity of plants as they enhance cell membrane integrity by reducing ROS and increasing tissue hydration status through osmotic adjustment [53, 54].

**Table 3:** Analysis of variance (two-way ANOVA) of the effect of *B. bassiana*, drought stress conditions and their interactions on some growth, biochemical parameters and stress markers in *M. parviflora* plants

		*p*-value	
Parameters	B. bassiana	Drought	B. bassiana * Drought
**Shoot length**	0.00^*^	0.00^*^	0.077^ns^
**Root length**	0.00^*^	0.00^*^	0.034^*^
**Shoot FW**	0.00^*^	0.00^*^	0.007^*^
**Root FW**	0.00^*^	0.00^*^	0.107^ns^
**Total Chl**	0.00^*^	0.00^*^	0.034^*^
**Total pigments**	0.00^*^	0.00^*^	0.048^*^
**Protein**	0.00^*^	0.00^*^	0.098^ns^
**TPC**	0.00^*^	0.00^*^	0.540^ns^
**TFC**	0.00*	0.00*	0.00*
**Proline**	0.00^*^	0.00^*^	0.016^*^
**AA**	0.001^*^	0.001^*^	0.024^*^
**CAT**	0.00^*^	0.00^*^	0.00^*^
**PAL**	0.00^*^	0.00^*^	0.00^*^
**TAC**	0.00^*^	0.00^*^	0.006^*^
**Shikimic acid**	0.00^*^	0.00^*^	0.00^*^
**MDA**	0.00^*^	0.00^*^	0.632^ns^
**H**_**2**_ **O**_**2**_	0.00^*^	0.00^*^	0.012^*^

* Significant at the *p* < 0.05; ns non-significant

Under drought and well-irrigated conditions, *B. bassiana* application efficiently increased the protein and carbohydrate content compared to non-inoculated plants. Under severe drought conditions, *M. parviflora* leaves bioprimed with *B. bassiana* showed 37.8, 19.5, and 65.3% increases in soluble sugars, amino acids, and proline compared to non-treated ones. As a result, they helped the inoculated plants cope with drought stress better. Drought tolerance noticed in *B. bassiana*-treated *M. parviflora* leaves compared to non-treated ones can be caused by increased solute accumulation, decreased leaf conductance, slower transpiration rates, or thicker cuticles [55]. Drought stress influences the plant-water relationship, causing complex plant responses, such as increased osmolytes production. The osmotic potential is primarily determined by solute potential and matrix potential; symbiotic fungi are likely to contribute to matrix potential, which is especially important in assisting plants in retaining water and improving plant drought tolerance because symbiotic fungi-associated plants significantly consume less water than non-symbiotic plants [33, 56].

**Fig. 5:**
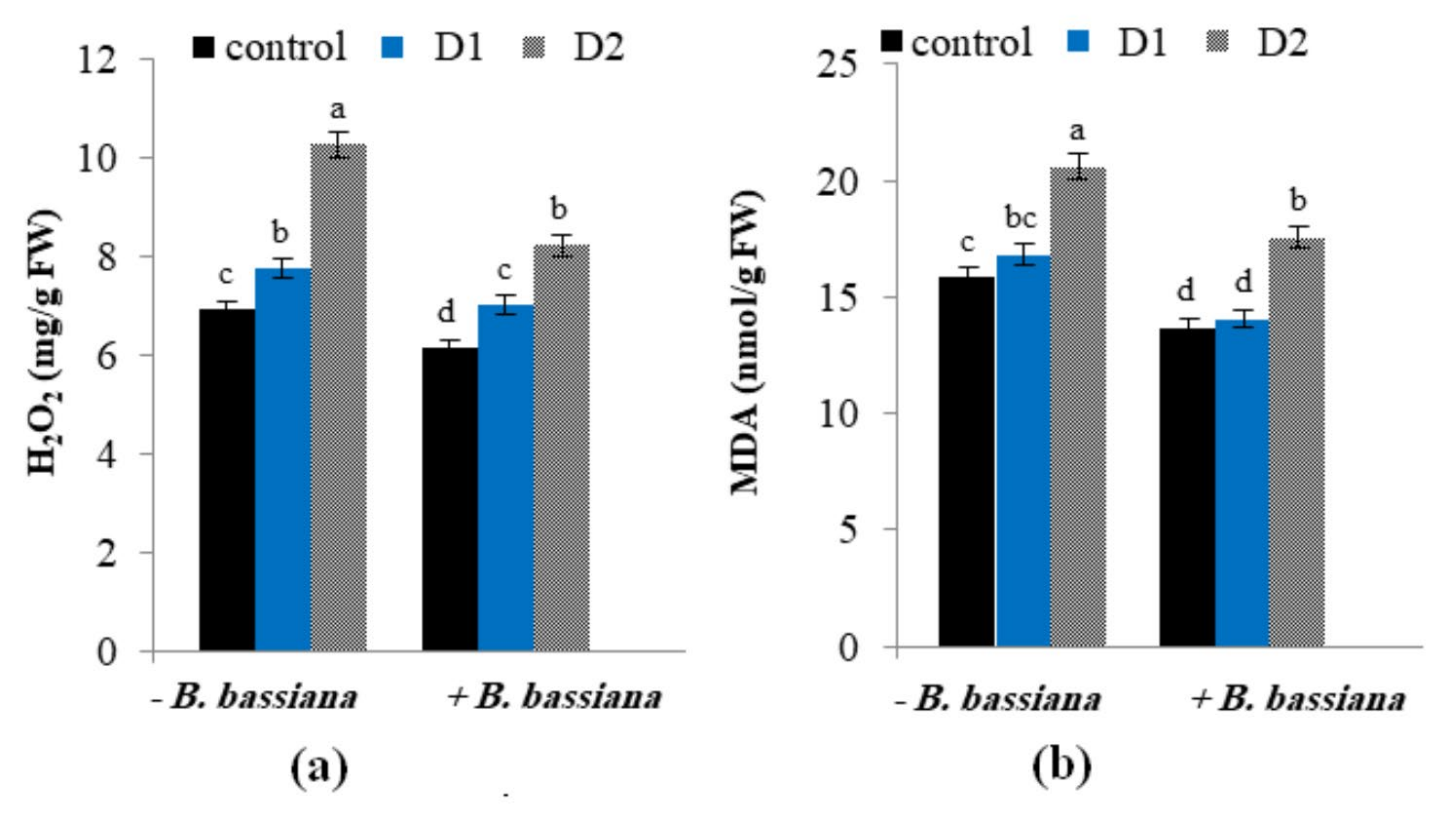
Effect of *B. bassiana* inoculation on the stress markers: (**a**) H_2_O_2_ and, (**b**) malondialdehyde (MDA) in *M. parviflora* L plants under drought stress. Data are the mean (*n* = 5). Error bars represent the standard error of the mean and different letters show significant differences at *p* < 0.05 according to One-Way ANOVA: Post Hoc Multiple Comparisons (Duncan)

These results align with Ghabooli and Kaboosi’s [57], which investigated tomato plants due to *Serendipita indica* inoculation. Aalipour et al. [58] reported an improvement in osmolytes in *Arizona cypress* treated with PGPR. The cellular osmotic potential was decreased by increasing osmolyte accumulation and permitting proper water absorption from the soil, which increased the cell turgor pressure, protected the water status of the cells, and preserved the membranes [59]. As a result of the application of endophytes to plant tissues, the quantity of osmolytes increases, which in turn increases resistance to water loss, protects chloroplasts, and promotes plant growth under stressful conditions. [55, 60, 61]. To maintain membrane integrity under drought conditions, *B. bassiana* boosted osmolytes, including soluble carbohydrates, proteins, and proline; cell membranes’ low level of peroxidation (less MDA) demonstrates this improvement (Fig. 5b).

### Influence of *B. bassiana* on oxidative stress markers (H_2_O_2_ and MDA) of drought-stressed *M. parviflora*

The overproduction of ROS, such as H_2_O_2,_ is one of the first metabolic reactions of plants in response to biotic and abiotic stressors [12, 62, 63]. The current study assessed the oxidative stress markers triggered by ROS generated in drought-stressed *M. parviflora* that cause lipid peroxidation. Therefore, H_2_O_2_ and MDA levels were determined (Fig. 5a, b). The obtained data showed that moderate and severe drought stress resulted in higher production of H_2_O_2_ (7.75 and 10.25 mg/g FW) and MDA levels (16.83 and 20.85 nmol/g FW) over control plants (6.91 mg/g FW and 15.87 nmol/g FW). The increase of H_2_O_2_ in *M. parviflora* demonstrated in the current study may have sped up the Haber-Weiss reaction, leading to the generation of the hydroxyl radical (^•^OH), which, in turn, caused severe lipid peroxidation and cell membrane damage [64]. The increase in MDA indicates that excessive H_2_O_2_ generation during drought stress results in pro-oxidative circumstances that may have caused cellular damage [62]. Likewise, Begum et al. [65] reported a more robust accumulation of H_2_O_2_ and MDA in tobacco plants under drought stress.

The beneficial effect of *B. bassiana* on *M. parviflora* appeared in the reduction in H_2_O_2_ and MDA contents compared to control plants grown under well-irrigated and drought-stress conditions (as shown in Fig. 5a, b; Table 4). Consequently, *B. bassiana* alleviated the oxidative stress in *M. parviflora*. Similar findings were previously determined in *Arizona cypress* and *Nicotiana tabacum* subjected to drought stress, in which less H_2_O_2_ and MDA contents were detected in plants treated with endophytes such as PGPR and mycorrhizal fungi [58, 65].

**Table 4:** Pearson’s correlation matrix between some growth, physiological, biochemical parameters and stress markers in *M. parviflora* plants. Each square indicates Pearson’s correlation coefficient of a pair of parameters

Measured parameters	Shoot length	Root length	Shoot FW	Root FW	Total Chl	Total pigments	Protein	Proline	MDA	H_2_O_2_
**Shoot length**	1	0.978**	0.971**	0.973**	0.979**	0.923**	0.996**	0.894**	-0.843**	-0.880**
**Root length**		1	0.982**	0.988**	0.983**	0.953**	0.975**	0.909**	-0.766**	-0.827**
**Shoot FW**			1	0.980**	0.963**	0.965**	0.965**	0.916**	-0.769**	-0.827**
**Root FW**				1	0.983**	0.990**	0.975**	0.935**	-0.835**	-0.875**
**Total Chl**					1	0.920**	0.990**	0.952**	-0.818**	-0.872
**Total pigments**						1	0.952**	0.847**	-0.611**	-0.726**
**Protein**							1	0.922**	-0.852**	-0.893**
**Proline**								1	-0.769**	-0.796**
**MDA**									1	0.965**
**H**_**2**_ **O**_**2**_										1

* Correlation was significant at *p* < 0.05

** Correlation was significant at *p* < 0.01

### Effect of *B. bassiana* on secondary metabolites of *M. parviflora* under drought stress

**Fig. 6:**
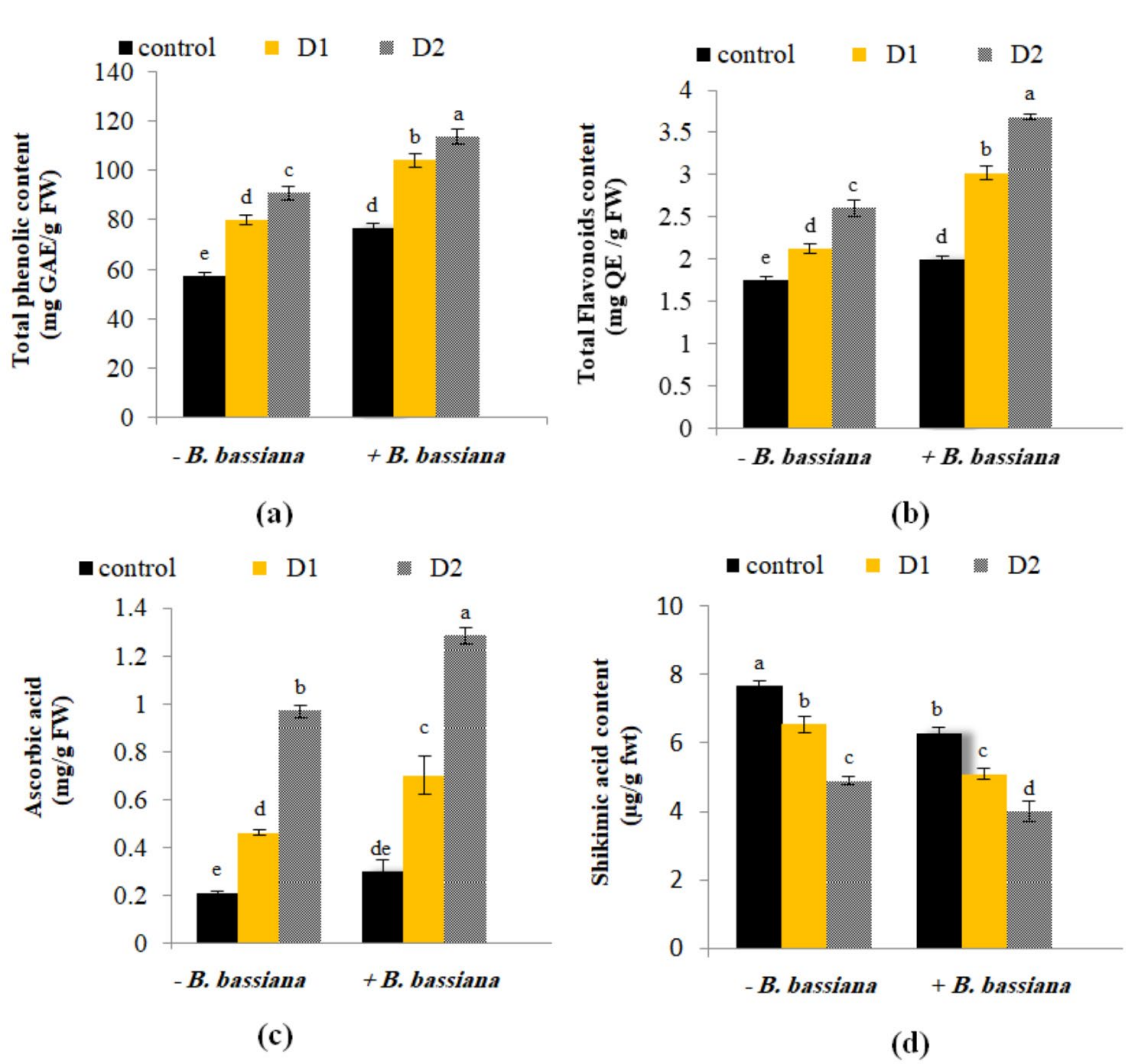
Effect of *B. bassiana* inoculation on the content of (**a**) total phenolic content, (**b**) total flavonoids content, (**c**) ascorbic acid (ASA) and (**d**) shikimic acid in *M. parviflora* L plants under drought stress. Data are the mean (*n* = 5). Error bars represent the standard error of the mean and different letters show significant differences at *p* < 0.05 according to One-Way ANOVA: Post Hoc Multiple Comparisons (Duncan). (GAE): gallic acid equivalent, (QE): quercetin equivalent

In the present study, TPC, TFC, and AA in *M. parviflora* leaves were increased under severe and moderate drought stress (Fig. 6a-c). This could result from the overproduction of ROS due to the drought stress. Preserving a balance between ROS production and scavenging is essential under stressed conditions. Sarker and Oba [64] indicated that water stress can cause an increase in ROS levels; therefore, higher antioxidant quantities are needed to counteract the stress effects. However, a further rise in their contents in *M. parviflora* leaves was recorded with *B. bassiana* application compared to non-treated plants (Fig. 6a-c). Gana et al. [1] found that the polyphenol content was increased in *Allium cepa* bulbs exposed to *B. bassiana*, an established source of beneficial secondary metabolites [66]. ROS in plants could encourage the production of more secondary metabolites, providing the necessary protection against stress. According to Venkatesh and Park [67], several plants overproduce ascorbate and demonstrate improved drought tolerance with reduced MDA and chlorophyll content loss.

Drought stress inhibits the conversion of shikimic acid to aromatic amino acids, causing shikimic acid to accumulate [68]. Our results showed an inverse relationship between shikimic acid concentration and the level of drought stress in non-bioprimed plants (Fig. 6d). Some drought-tolerant species or variants can maintain the standard conversion of shikimic acid by activating alternative metabolic pathways [69]. Correia et al. [70] affirmed a decrease in the concentration of shikimic acid in drought-stressed *Eucalyptus globulus*, while an increase was observed in response to heat stress. Similarly, Hochberg et al. [71] and Tamayo et al. [72] found that shikimic acid was downregulated in plant tissues under water-deficit treatment. Drought stress triggers an increase in enzymes involved in flavonoid and stilbene biosynthesis in the roots of grapevine rootstock [73], which decreases shikimic acid content. In addition, the contents of metabolites in the shikimate pathway (i.e., quinic and shikimic acids) were substantially reduced in maize plants under conditions of drought and combined drought and salt stress [74]. Thus, while shikimic acid accumulation is the typical response to drought, reductions are possible contingent on the plant’s drought tolerance mechanisms, timing, and severity.

Remarkably, *B. bassiana* inoculation significantly decreased the concentration of shikimic acid compared to non-inoculated plants (Fig. 6d). Besides, under moderate and severe drought, the level of shikimic acid was significantly lower (*p* > 0.05) in bioprimed plants than non-primed plants. The synthesis of protective secondary metabolites, which include aromatic amino acids (phenylalanine, tyrosine, and tryptophan) [75] and phenolic through the shikimate/phenylpropanoid pathway [76], could be the cause of the decreased level of shikimic acid in primed plants. These compounds participate in various physiological processes in plants and serve as an adaptive response to unfavorable environmental conditions.

### Effect of *B. bassiana* on enzymatic antioxidant machinery in drought-stressed *M. parviflora*

**Fig. 7:**
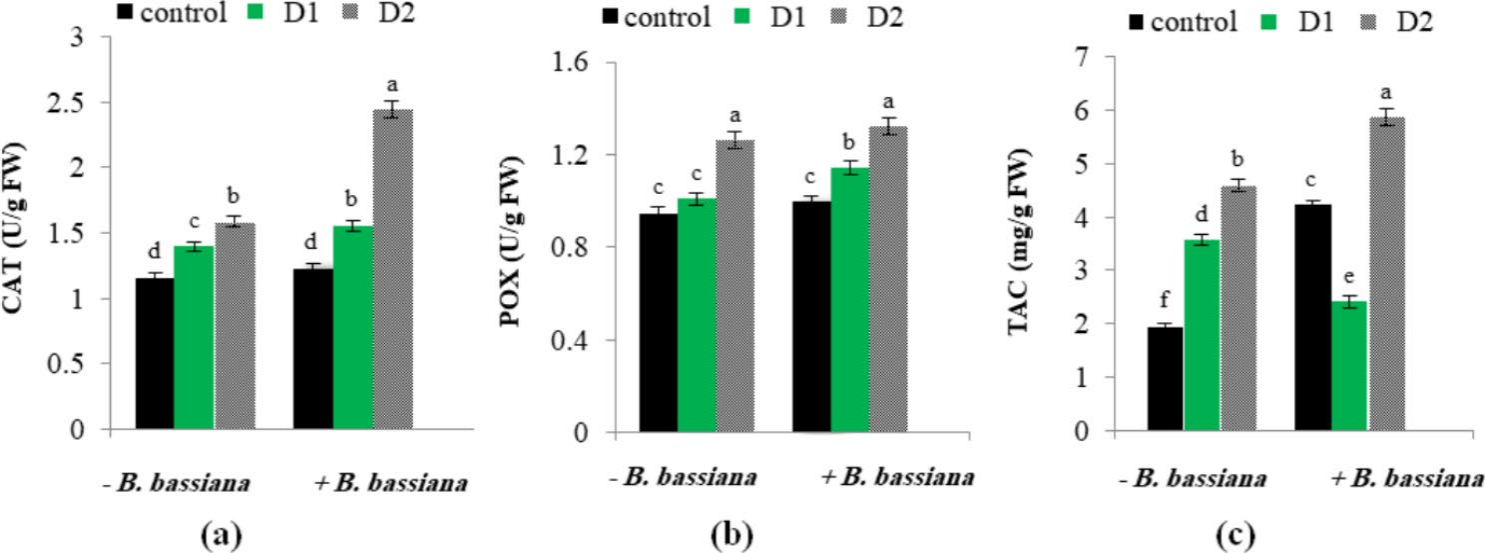
Effect of *B. bassiana* inoculation on the antioxidant: (**a**) CAT, (**b**) POX and (**c**) TAC in *M. parviflora* L plants under drought stress. Data are the mean (*n* = 5). Error bars represent the standard error of the mean and different letters show significant differences at *p* < 0.05 according to One-Way ANOVA: Post Hoc Multiple Comparisons (Duncan)

Numerous enzymatic antioxidant systems are present in plants to protect them from the damage caused by oxidative stress. [63, 77]. The findings in Fig. (7a–c) demonstrated that TAC and the evaluated enzymes were upregulated in *M. parviflora* leaves due to water shortage. Compared to plants cultivated under controlled circumstances, severe drought stress dramatically boosted CAT and POX activities by 36.1 and 33.28%, respectively. In contrast, treatment of *M. parviflora* with *B. bassiana* under severe drought stress increased the CAT and TAC activities by 54.2 and 27.8% compared to those non-inoculated ones. By modifying the antioxidant activities and detoxifying ROS, these findings clarify the favorable effect of *B. bassiana* inoculation on increasing drought tolerance in *M. parviflora*. A similar finding was observed in drought-stressed *M. oleifera* plants treated with endophytic consortia with significantly higher antioxidant enzyme activities than stress-induced control plants [11].

**Fig. 8:**
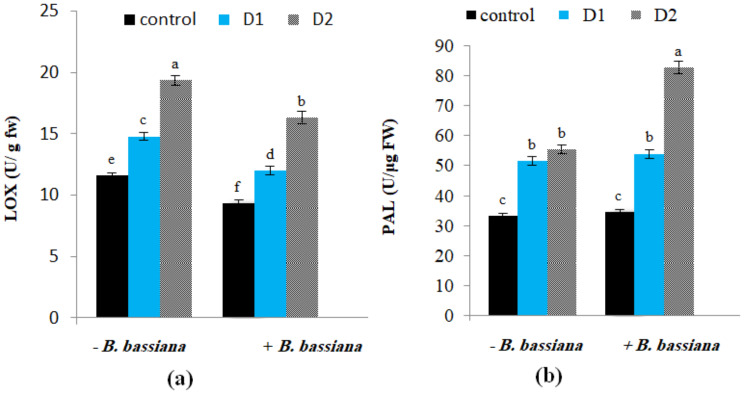
Effect of *B. bassiana* inoculation on the activity of (**a**) lipoxygenase (LOX), and (**b**) phenylalanine ammonia-lyase (PAL) in *M. parviflora* L plants under drought stress. Data are the mean (*n* = 5). Error bars represent the standard error of the mean and different letters show significant differences at *p* < 0.05 according to One-Way ANOVA: Post Hoc Multiple Comparisons (Duncan)

LOX and PAL activities were affected by *B. bassiana* application and drought stress in *M. parviflora*. An increase in LOX activity under moderate and severe drought conditions was detected (Fig. 8a). In line with the present results, Rezayian et al. [78] monitored a significant increase in LOX activity in polyethylene glycol-stressed *Glycine max* plants compared to the control. On the contrary, *B. bassiana* inoculation decreased LOX activity in *M. parviflora* under both normal and drought-stressed conditions compared to non-bioprimed control. LOX is considered a trigger of adaptive responses to stress and a marker of damage; it causes the oxidation of polyunsaturated fatty acids and enhances lipid peroxidation and membrane deterioration under stress [79]. As a result, its decrease is caused by *B. bassiana* application, which inhibits the peroxidation of membrane lipids, protecting membrane integrity and enhancing drought stress tolerance in *M. parviflora*.

The present results also showed an increase in PAL activity that reached 45.8 and 66.2% under moderate and severe drought conditions over the corresponding control (Fig. 8b). Similarly, Gholizadeh [80] noticed a sharp increase in PAL activity in maize leaves during drought stress. Furthermore, *B. bassiana* inoculation promotes PAL activity under normal conditions, with further enhancement in its content under drought compared to the non-inoculated plants. Da Trindade et al. [81] showed that endophytes enhanced PAL enzyme activity in *Piper nigrum*, an essential enzyme in the phenylpropanoid pathway that catalyzes the production of crucial phenolic compounds such as lignins, flavonoids, and phytoalexins, functioning as antioxidants, reinforcing cell walls, and aiding in stress tolerance and adaptation [82].

### Effect of *B. bassiana* on K and Mg accumulation in *M. parviflora* under drought stress

**Fig. 9:**
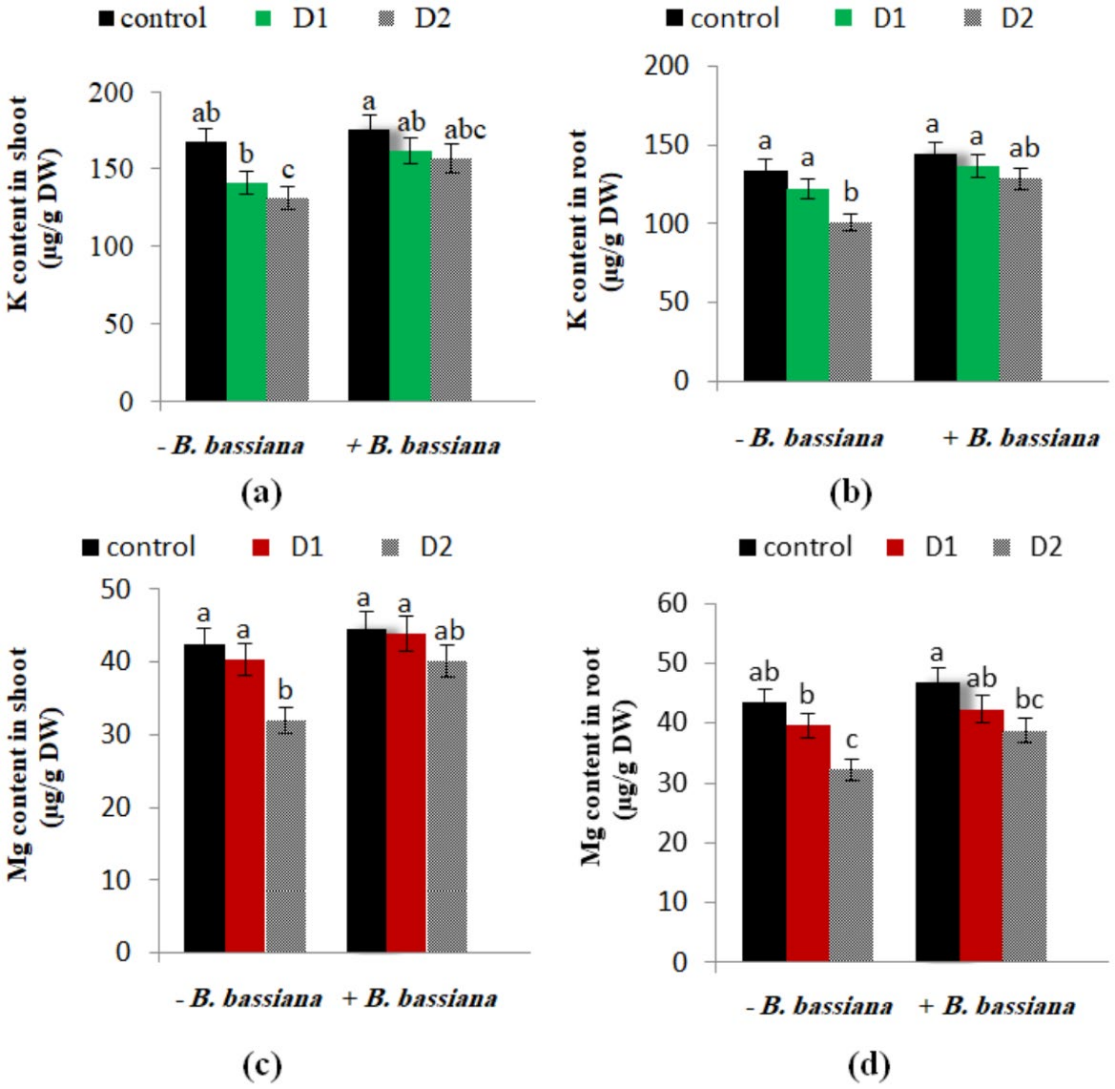
Effect of *B. bassiana* inoculation on the mineral contents (**a** and **b**) K and (**c** and **d**) Mg in shoot and root of *M. parviflora* L plants under drought stress. Data are the mean (*n* = 5). Error bars represent the standard error of the mean and different letters show significant differences at *p* < 0.05 according to One-Way ANOVA: Post Hoc Multiple Comparisons (Duncan)

K and Mg contents of *M. parviflora* were found to be progressively influenced by drought stress. The contents of K and Mg in *M. parviflora* shoots and roots are shown in Fig. (9a-d). K and Mg contents decreased under drought stress, whereas with *B. bassiana* application, a remarkable increase in their contents in both shoots and roots of *M. parviflora* was observed. In agreement with our findings, Zhang et al. [83] found that drought stress significantly affects the uptake of nutrients, photosynthetic activity, and seedling germination in *Zenia insignis*, leading to a general decline in growth. Selvakumar et al. [84] noted that drought stress impacts the availability and movement of soil nutrients, as water transports these nutrients to the roots, consequently decreasing nutrient diffusion and mass flow of water-soluble nutrients, such as Ca and Mg. Additionally, *Sporormiella intermedia* endophyte improved Ca, Zn, and Cu absorption in the subclover [85]. Although micronutrients were remarkably enhanced by *B. bassiana*, Macuphe et al. [18] found no significant change in N, P, K, Ca, and Mg contents between *B. bassiana*-treated lettuce and control plants. The discrepancies between our findings and those reported by Macuphe et al. [18] are challenging to explain, but they may be due to variations in several experimental factors. For instance, differences in plant growth conditions, plant species utilized, and endophyte treatment inoculation methodologies may justify the two studies’ divergent results. During drought stress, Ca, Mg, P, and N levels in *A. cepa* bulbs were elevated in *B. bassiana*-treated plants, confirming that endophytes improve macronutrient absorption to lessen the negative impacts of water stress [1]. Furthermore, Chakraborti et al. [86] stressed the role of endophytes in converting unavailable nutrients into bioavailable forms and their sequestration into plants. These nutrients are essential for photosynthesis, cell membrane integrity, protein synthesis, stress resistance, and the augmentation of antioxidant enzymes and chlorophyll levels in plant tissues [1].

### Effect of *B. bassiana* on the hormonal content of *M. parviflora* under drought stress

**Fig. 10:**
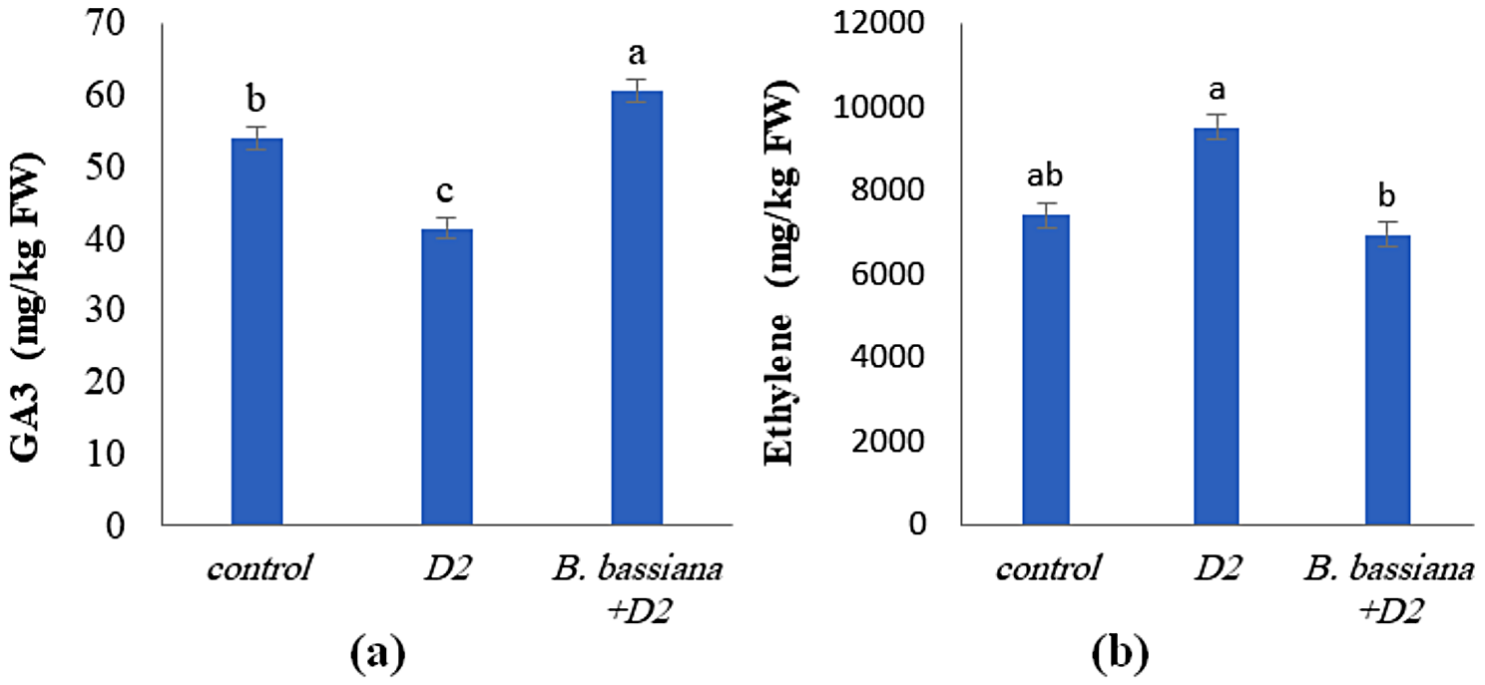
Effect of *B. bassiana* inoculation on the hormone concentration; (**a**) Gibberellic acid; GA3 and (**b**) ethylene in *M. parviflora* L plants under drought stress. Data are the mean (*n* = 5). Error bars represent the standard error of the mean and different letters show significant differences at *p* < 0.05 according to One-Way ANOVA: Post Hoc Multiple Comparisons (Duncan)

Figure (10a, b) demonstrates that *B. bassiana* significantly altered the level of hormones (GAs and ethylene) produced in *M. parviflora* and attenuated the harmful effects of drought stress. Comparing drought-stressed *M. parviflora* to controls revealed a decrease in their GAs content (Fig. 10a). Likewise, Wang et al. [87] found a reduction in GAs content in maize leaves due to water stress. Previous research showed that drought and osmotic stress in *Arabidopsis thaliana* resulted in changes in gene expression, expected to reduce GA content [88].

Moreover, with *B. bassiana* application, an increase in GAs content was detected under drought stress (Fig. 10a). Proietti et al. [89] discovered that various active GAs and GA precursors, such as GA9 and GA20, were highly up-regulated in *B. bassiana*-colonized tomato plants compared to controls. Furthermore, specific proteins up-regulated by *B. bassiana* colonization could be associated with the gibberellin pathway. For example, NADPH-cytochrome P450 reductase is involved in manufacturing various secondary metabolites, including GAs [90]. Another one is the ‘C_2_H_2_-type domain-containing protein,’ which is involved in forming GAs [91]. This finding could explain the observed GAs buildup after *B. bassiana* colonization. According to Verma et al. [33], fungal endophytes such as *Penicillium* sp. inhabiting *Suaeda japonica* secrete PGP compounds such as GAs under stress. As reported previously, drought tolerance in plants is noticeably enhanced by GAs [92], which act as growth hormones and provide resistance against drought stress and other abiotic stresses. GAs strengthen the development of plant tissues through cell elongation and increase the cell division process, subsequently improving the immature and adult stages of plant growth. They also help to augment plants’ vegetative and reproductive stages [93].

Ethylene can positively or negatively regulate abiotic stress tolerance [94]. Concerning the effect of drought stress on ethylene content, Fig. 10b highlights a significant increase in its content under severe drought stress (9491 mg/kg FW) compared to control (7397 mg/kg). When ethylene is present in substantial amounts, especially under stressful circumstances, it harms plant development and survival because it accelerates plant senescence and causes other stress-related symptoms [95, 96]. Drought amplifies ethylene production, which hinders plant development through various mechanisms. Some adverse effects include heightened leaf shedding, reduced stomatal activity, inhibited root and shoot growth, leaf curling, prolonged root system hypoxia, cellular damage, and eventual plant death [97].

Moreover, inoculation with *B. bassiana* decreased ethylene content (6939 mg/kg FW) with a non-significant value compared to the control. These findings correspond to Goswami and Suresh’s [98] that PGPR causes plants to activate defense mechanisms such as enhanced antioxidant metabolism, osmotic adjustment, and ethylene inhibition, all of which support the plant’s immune system and defenses against abiotic stressors. Reducing ethylene levels decreases plant stress and enhances growth and development [86]. *B. bassiana’*s regulation of ethylene synthesis could maintain growth and provide a rapid indication for responding to drought-stress conditions.

### Effect of *B. bassiana* on genetic variation of the *M. parviflora* under drought stress

**Fig. 11:**
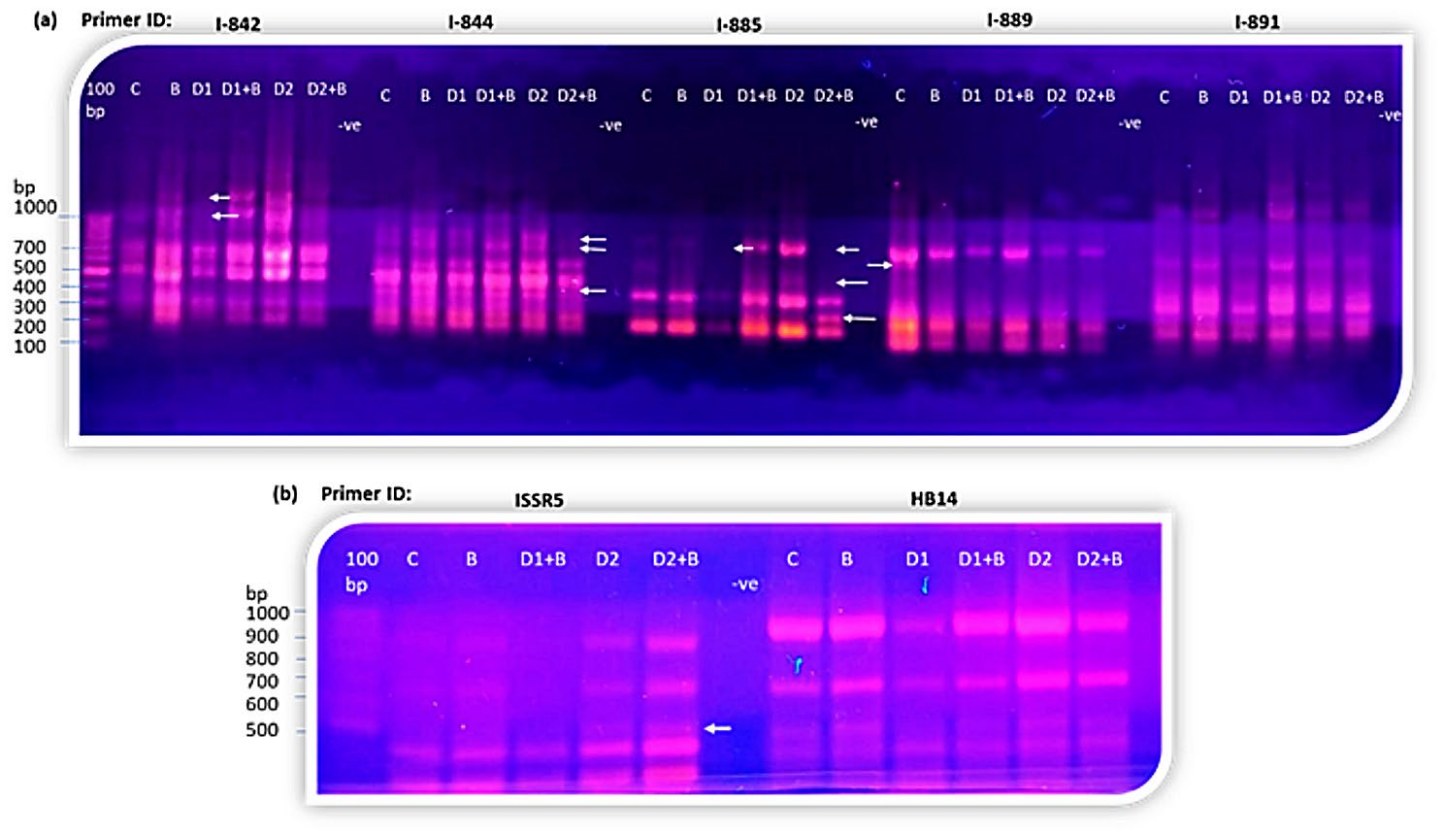
Photographs illustrating ISSR fingerprints amplified from *M. parviflora* L plants’ DNA in response to different treatments by seven different primers: (**a**) shows the electrophoretic pattern for I-842, I-844, I-885, I-889 and I-891 primers. (**b**) shows electrophoretic pattern for ISSR5 and HB14 primers. 100 pb refers to the DNA ladder, -ve refers to negative control and no amplification confirms no PCR contamination. Arrows refer to polymorphic bands

Exploring stress response and tolerance mechanisms has commenced by identifying their genetic alterations. The extent of genetic variation in stressed and unstressed plants would determine the plant’s response to the applied stress. ISSR, Scot, and SNP genome markers were utilized for high-throughput analyses of genetic variation in plants [9, 99–101]. The generation of genetic diversity is crucial for genetic enhancement, facilitating increased genetic gains and enhanced performance in subsequent generations of plants that experience stress. Ten ISSR primers were used to investigate genetic variability induced by different drought and/or *B. bassiana* treatments in *M. parviflora* plants. Only eight primers amplified 40 consistent markers satisfactorily, thirty were monomorphic, and ten were polymorphic (Table 5; Fig. 11). The amplified markers varied in size from 5053 to 189 base pairs, with a total polymorphism of 25%. I-885 exhibited the highest level of polymorphism (60%), whereas I-812, I-891, and HB14 exhibited no variability. Small numbers of ISSR markers were scored for each treatment, with a maximum of seven markers amplified by primer I-844 and only four by primers I-812, I-889, and HB14.

**Table 5:** List of selected ISSR primers, including their codes, sequences, annealing temperature, number of amplified markers, and percentage of polymorphism for each primer in each treatment

No.	Primers codes	Sequencing (5′-3′)	Annealing temp. (°c)	No. of Polymorphic markers	Total no. of markers	% of polymorphism
1.	I-868	(GAA)_5_	50			
2.	I-827	(AC)_8_G				
3.	I-842	(GA)_8_CTG		2	6	33.33
4.	I-812	(GAG)_5_AT		0	4	0
5.	I-844	(CT)_8_GC		3	7	42.8
6.	HB14	(GT)_6_CC		0	4	0
7.	I-885	CGTACTCGT(GA)_5_		3	5	60
8.	I-889	AGTCGAGT(AC)_5_		1	4	25
9.	I-891	ACTACGACT(TG)_5_T		0	5	0
10.	ISSR-5	(ACG)_4_GAC		1	5	20
				10	40	25

**Fig. 12:**
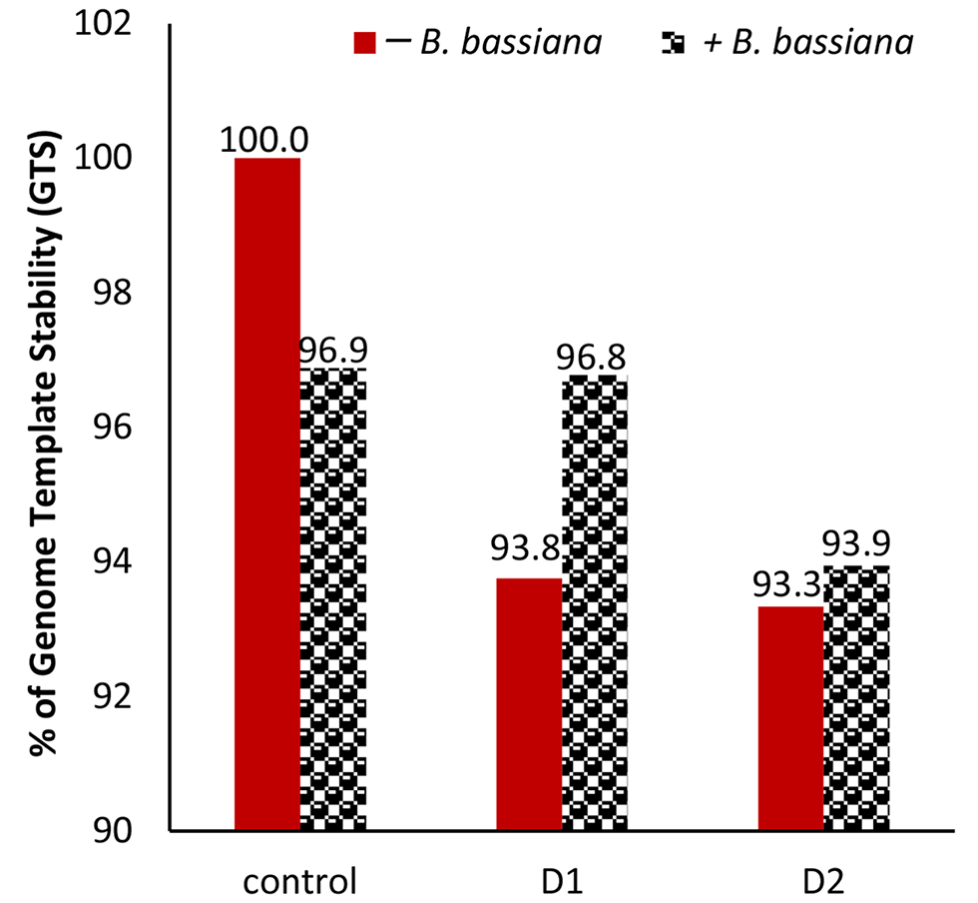
Comparison of genomic DNA template stability (GTS) in *M. parviflora* plants in response to drought and/or *B. bassiana* treatments and the control

The percentage of genomic DNA template stability (GTS) for each treatment was used to calculate the diversity in the ISSR profile produced by various drought and/or *B. bassiana* treatments on the genetic composition of *M. parviflora* plants (see Fig. 12). In all treatments, the percentage of GTS was reduced to some extent. Both drought treatments slightly reduced the GTS to ∼ 93%, while the inoculation with *B. bassiana* for drought-treated plants retained the genome stability. The positive effect of *B. bassiana* inoculation in GTS was more effective for moderate drought treatment, reaching ∼ 97% compared to ∼ 94% for severe drought treatment.

The alterations in the ISSR profile show that drought stress and *B. bassiana* biopriming contributed to genetic variation. The appearance of new DNA bands and the absence of typical ones in the ISSR profile can promote the physiological and biochemical parameters of *M. parviflora* in response to biopriming or decrease them in response to drought. These bands possibly come from the genomic variation caused by dehydration and/or biopriming, which prevents mitosis and alters DNA by causing chromosomal abnormalities. Wheat seedlings underwent destabilization and unequal cell content and chromosome distribution due to salt stress [102]. The induced genetic variation, the decreased GTS percentage in drought-stressed plants, and the retained GTS by *B. bassiana* biopriming can be explained by their indirect interaction with nuclear proteins and/or DNA influenced by oxidative stress induced by ROS. Oxidative stress markers such as MDA and H_2_O_2_ were elevated in drought-treated plants, whereas *B. bassiana* decreased these stress markers. Besides, oxidative stress induces the production of free radicals that interact with DNA, causing its degradation and interfering with the cell cycle, resulting in genome modification, thereby compromising the DNA repair mechanisms [103]. The formation of an abnormal tubulin cytoskeleton in plant cells is a common and non-specific response to various ROS generated by abiotic stresses, leading to an aberrant cell cycle [104, 105]. Previous research has demonstrated that drought stress affects genome stability and causes genetic variation in the grassland species *Bouteloua eriopoda* [106].

## Conclusion

Drought stress resulted in decreased growth, physiology, genome stability, and ionic attributes of *M. parviflora* due to a decrease in Chl content and impaired water status. However, priming with *B. bassiana* improved the growth, genome stability, and physio-biochemical parameters of *M. parviflora* by enhancing the accumulation of enzymatic antioxidants and secondary metabolites, which resulted in improved Chl content and leaf water status with a concomitant decrease in lipid peroxidation and H_2_O_2_. The key findings demonstrated that *B. bassiana* inoculation reduces the adverse effects of drought stress on plant growth by up-regulating the synthesis of secondary metabolites (TPC, TFC, and AA), concomitant with genome rearrangement in *M. parviflora* that becomes apparent under severe drought stress. Furthermore, the findings showed that priming with *B. bassiana* increased K and Mg levels, phytohormone (GAs) content, and reduced ROS that may interact with DNA, causing genome modification to compromise the drought stress effects. These changes due to *B. bassiana* were reflected in the growth and secondary metabolism of *M. parviflora*. By incorporating *B. bassiana*, *M. parviflora’s* nutritional value can be increased, and yield losses in places with limited water supplies could be decreased.

## Materials and methods

### Procurement of *B. bassiana*

*Beauveria bassiana* strain RA1 was obtained from the fungal collection of the Assiut University Mycology Center, Assiut University, Egypt and deposited under No. AUMC 9895. *B. bassiana* was grown on Potato dextrose agar (PDA) media in Petri dishes (90 mm.) at 23–27 °C for 10 days. Its hyphae, conidia, and conidiophores were observed and photographed with a compound microscope.

### PCR amplification and phylogenetic analysis

For molecular identification, the pure culture of *B. bassiana* was sub-cultured on a PDA medium and grown for 7 days at 25 °C and its genomic DNA was isolated using the CTAB technique after breaking down the cell walls of its mycelia by liquid N_2_ [107]. The CTAB extraction buffer was added, and the mixture was incubated at 65 °C. Finally, the DNA was dissolved in 50 µL of sterilized distilled water.

Using the ITS1 and ITS4 primers, the ITS1 and ITS2 and the interspaced 5.8 S coding rDNA were amplified according to White et al. [108]. Before DNA sequencing, the PCR amplicon was resolved using 0.8% agarose gel and purified using a specific PCR purification kit (Accu Prep^®^ PCR DNA Purification Kit, K-3034-1, Bioneer Corporation, South Korea). Macrogen Inc, (South Korea) sequenced the purified PCR products on both strands of the submitted DNA using an ABI 377 DNA auto sequencer (PerkinElmer, Applied Biosystems Div., Waltham, USA) based on the same primers mentioned before.

### Preparation of spore suspension

To achieve complete sporulation, the *B. bassiana* RA1 strain was prepared from a stock culture in Petri dishes (90 × 15 mm) with 20 mL of PDA. It was then maintained in darkness at 23 °C for 10 days. Conidia were then scraped, harvested, and suspended in sterile distilled water before being centrifuged at 3.000 rpm for three min to remove hyphal fragments, conidial clumps, and agar [109]. The sterilized *M. parviflora* seeds were bioprimed with *B. bassiana* RA1 at a concentration of 2 × 10^7^ conidia/mL distilled water.

### Plant materials, sterilization and priming

*M. parviflora* seeds were attained after permission from the Department of Agricultural Research Center, Giza, Egypt and surface sterilized for 5 min with 1.5% sodium hypochlorite (NaOCl), rinsed three times with autoclaved distilled water, and dried under laminar air flow [110]. The surface sterilized seeds were divided into two sets. The first set was treated by soaking the seeds in *B. bassiana* spore suspensions (2 × 10^7^ conidia/mL) and the other set (control) was primed with sterile water for 24 h. The two sets of seeds were dried separately in laminar air flow for 2 h, and then put in a moist chamber where they were kept at 28–30 °C and 98% relative humidity for 24 h [111].

### Pot trial, treatment detail and experimental design

The *B. bassiana* primed and hydro-primed *M. parviflora* seeds were sown in plastic pots (15 cm diam.) containing 2 kg of sterilized soil from an agricultural field located in Minia Al-Qamh, El-Sharkia Governorate. Under the controlled environment of the plant growth chamber of the Faculty of Science, Zagazig University, Egypt (light duration of 11 h and dark 13 h with a relative humidity range of 50–70% and the temperature ranging from 22 to 25 °C, while night temperature was maintained at 18 °C), the experiment was carried out. Ten seeds per pot were sown and irrigated regularly. The pots were thinned after germination to 5 seedlings/pot. After 10 days from germination, *M. parviflora* plants in the *B. bassiana* treatment received an additional 100 mL of conidial suspension, whereas the control plants received 100 mL of sterile distilled water.

### Irrigation and drought stress application

For drought application, each set of *M. parviflora* plants (*B. bassiana* primed and hydro-primed) was differentiated into 3 groups, thus the experiment had a fully randomized design with factorial planning of 2 × 3. Each treatment had five replicates (*n* = 5), and the total number of pots in the study was 30. For irrigation purposes, tap water was used. After 20 days from germination, the drought stress was administered at three different water regimes, i.e., controlled normal irrigation (90% WHC “water holding capacity”), mild drought stress (60% WHC), and severe drought stress (30% WHC) based on Piper [112].

### Measurements

#### Harvesting and data collection

*M. parviflora* plants were collected from each treatment after 50 days from germination (30 days from drought application). All plant roots were rinsed with distilled water to remove soil particles. Fresh weights (FW) were measured immediately. Shoot heights and root lengths were measured. Fresh plant samples were stored in a biomedical freezer (-10 °C ~-25 °C) for further biochemical analysis. Samples were oven-dried for 72 h at 65 °C and were used to measure their dry weights (DW) and ion analysis.

### Colonization of *M. Parviflora* roots by *B. Bassiana*

*M. parviflora* roots were carefully taken from randomly selected plants from each treatment. After surface sterilization, *M. parviflora* plants were rinsed with tap water for 5 min, submerged in 70% ethanol for 10 s, and then washed in sterile distilled water for 5 min. The efficacy of fungal colonization was evaluated by plating 1–2 cm long segments of *M. parviflora* roots on PDA plates containing 0.04 g streptomycin and 0.02 g ampicillin sodium salt. They were then incubated for 14 days in the dark at 25 °C.

### Physio-biochemical attributes of *M. parviflora* plants under drought-stressed conditions

#### Chlorophyll contents

After extraction in 10 mL of 85% acetone, the chlorophyll (Chl.) a, Chl b, and carotenoids of *M. parviflora* [113] were estimated in a known FW (0.1 g). Light absorbance was recorded respectively, at 644, 663, and 452.5 nm using a UV-visible spectrophotometer, RIGOL (Model Ultra-3660).

### Measurement of *M. parviflora* water status

*M. parviflora* leaves were collected from the different treatments and their FW was measured individually. They were subsequently dipped in distilled water for about 4 h. After that, their turgid weight (TW) was measured individually. The leaves were then placed in an oven at 65 °C for 72 h to dry them and then their DW was determined [114]. The following equations were used to compute the water content (WC), relative water content (RWC), and water saturation deficit (WSD) of *M. parviflora* leaves:

$${\rm{RWC}}\left( {\rm{\% }} \right)\,{\rm{ = }}\,{{{\rm{(FW - DW)}}} \over {{\rm{(TW - DW)}}}}\,{\rm{X}}\,{\rm{100}}$$

$${\rm{WC}}\left( {\rm{\% }} \right)\,{\rm{ = }}\,{{{\rm{(FW - DW)}}} \over {{\rm{FW}}}}\,{\rm{X}}\,{\rm{100}}$$

$${\rm{WSD}}\left( \% \right) = 100 - {\rm{RWC}}$$

### Osmolytes determination (total soluble protein, proline content, sugars and total free amino acids)

The total soluble protein content of *M. parviflora* fresh leaves (0.25 g) was determined at A_700_ nm [115] after grinding with 5 mL of 1 N NaOH. The homogenate was then centrifuged at 6000 rpm for 30 min at 4 °C. Combined with a freshly prepared alkaline copper solution, 1 mL of solubilized protein was left for 10 min, followed by the addition of Folin-Ciocalteau reagent for 30 min. Also, proline contents in *M. parviflora* leaves were measured after being homogenized with 3% sulphosalicylic acid [116]. The optical density of the supernatant (2 mL) was measured at 520 nm after the addition of ninhydrin and glacial acetic acid. To extract 0.1 g of dried *M. parviflora* leaves, 2.5 N HCl was used to quantify the total amount of carbohydrates and 5% phenol and concentrated H_2_SO_4_ were combined with one mL of the extract [117], and the absorbance was measured at A_490_ nm. The amino acid contents in an alcoholic extract of *M. parviflora* leaves were determined [118] at A_570_ nm. Half mL of the extract was added to equal volumes of ninhydrin solution and citrate buffer (0.5 M, pH 5.6), and 1.2 mL of glycerol was added to them. Amino acids content was calculated as mg/g FW by referring to a standard curve prepared from glycine.

### Estimation of stress biomarkers [hydrogen peroxide (H_2_O_2_) and malondialdehyde (MDA) contents

H_2_O_2_ levels in *M. parviflora* leaves were determined and measured at A_390_ nm after homogenizing in 2 mL of 0.1% TCA [119]. The amount of H_2_O_2_ was calculated using (1, 5 and 10 mM H_2_O_2_) standard solutions and expressed as mg/ g FW. Also, MDA accumulation in *M. parviflora* fresh leaves was measured by thiobarbituric acid (TBA) reaction [120] after homogenizing with 2.0 mL of 20% trichloroacetic acid (TCA) (w/v) containing 1% TBA (w/v) for 30 min at 95 °C. The reaction product was measured at A_532_ nm and the quantity of MDA was calculated as nmol/g FW.

### Assay of non-enzymatic antioxidants of *M. Parviflora*

Ascorbic acid (AA) (Vitamin C) of *M. parviflora* fresh leaves (0.2 g) was estimated at A_540_ nm after extraction with 4% TCA [121]. The resultant ascorbate extract was added to Dinitrophenylhydrazine (DNPH) reagent (0.5 mL), followed by 2 drops of 10% thiourea solution. The contents were incubated at 37 °C for 3 h, resulting in the formation of osazone crystals that were dissolved in 2.5 mL of 85% H_2_SO_4_. The concentration of AA in the samples was calculated and expressed in terms of mg/g FW.

Following a 95% ethanol extraction, the total phenolic content (TPC) of *M. parviflora* leaves was quantitatively determined at A_650_ [122]. After that, 1 mL of the extract was combined with the Folin reagent and 20% Na_2_CO_3_. TPC was determined using the pyrogallol standard curve. Additionally, the aluminum chloride colorimetric test was used to quantify the total flavonoid content (TFC) [123]. At 510 nm, absorbance was measured. TFC was calculated from the quercetin equivalent (QE) standard curve and represented as mg QE/g FW for *M. parviflora* leaves. Also, the shikimic acid content was determined at A_380_ nm [124] after homogenizing in 0.25 M HCl and then centrifuging. The supernatant (50 µL) reacted with 1% periodic acid for 3 h at room temperature, then 0.5 mL of 1 M NaOH and 0.3 mL of 0.1 M glycine were added. The shikimic acid content was expressed as µg/ g FW.

### Extraction and assay of antioxidant enzyme activities

*M. parviflora* fresh leaves (1 g) applied or not with *B. bassiana* under well-irrigated or drought stress conditions were extracted using 10 mL 50 mM phosphate buffer pH 7.0 containing 0.1 mM EDTA and 1% polyvinyl pyrrolidone [125]. At 4 °C, the homogenate was centrifuged for 10 min at 8000 rpm.

The catalase (EC 1.11.1.6; CAT) activity in *M. parviflora* leaves was determined [126] by the consumption of H_2_O_2_ at A_240_ nm for 2 min. Also, peroxidase ((EC 1.11.1.7; POX) activity was assayed by determining the increase of absorbance at 470 nm with pyrogallol as the substrate [127].

The phosphomolybdenum technique was used to measure the total antioxidant capacity (TAC) of methanolic extracts of *M. parviflora* leaves [128]. A 3 mL reagent solution was combined with 1 mL of the extract (0.6 M H_2_SO_4_, 28 mM sodium phosphate, and 4 mM ammonium molybdate). The mixture was heated to 95 °C for 90 min, cooled, and the absorbance at 695 nm was determined.

### Assay of LOX and PAL activities

By measuring the absorbance at A234 nm, lipooxygenase (EC 1.13.11.12; LOX) activity in *M. parviflora* extract was determined [129]. The LOX activity was expressed as U/g FW, and the reaction was initiated by adding the crude extract to 2 mL of a 50 mM sodium phosphate buffer (pH 7.5) containing 1 mM of linoleate.

According to McCallum and Walker [130], the phenylalanine ammonia lyase (EC 4.3. 1.24; PAL) activity of *M. parviflora* enzyme extract was measured using an adaption of Zucker’s [131] method. L-phenylalanine was added to a 0.06 M borate buffer and crude enzyme to start the reaction. For 30 min, tubes were incubated. PAL activity was estimated by measuring A_290_ of the supernatant.

### K and Mg contents

Shoots and roots of *M. parviflora* were oven-dried and digested in a mixture of H_2_SO_4_: H_2_O_2_ (2:1 v/v) [132] using a wet digestion protocol. The samples were filtered and diluted with distilled water. Potassium (K) and magnesium (Mg) contents were determined spectrophotometrically at the Faculty of Veterinary Medicine’s Central Lab, Zagazig University, using Inductively Coupled Plasma Spectrometry (ICPS). K and Mg concentrations were calculated [133] according to the following equation:

$${\rm{Concentration = }}{{{\rm{ICPS}}\,{\rm{reading}}\,{\rm{x}}\,{\rm{total}}\,{\rm{volume}}\,\left( {{\rm{mL}}} \right)} \over {{\rm{Weight}}\,{\rm{of}}\,{\rm{sample}}\left( {\rm{g}} \right)}}$$

### Hormonal analysis (gibberellic acid; GAs and ethylene)

Fresh *M. parviflora* leaves weighing ten grams were blended with 70% methanol and swirled for an entire night at 4 °C. The extract was filtered, and under a vacuum, the methanol evaporated. The resulting aqueous phase was partitioned three times with ethyl acetate after being adjusted to pH 8.5 using a 0.1 M phosphate buffer. The aqueous phase was pH-adjusted to 2.5 with 1 N HC1, then partitioned three times with basic ethyl acetate fraction afterward. The hormone-containing dry residues from the acidic and basic ethyl acetate fractions were dissolved in methanol and kept at 4 °C after being passed through anhydrous sodium sulphate and evaporated under vacuum.

The extract was diluted in 1 mL of analytical-grade methanol, and the phytohormones were identified using high-performance liquid chromatography (HPLC) analysis. A model (HP 1050) HPLC with a UV detector was used for analysis at the Agricultural Research Center (Soils, Water and Environment Res. Inst. “SWERI”), Egypt. To separate and determine, a C18 column (4.6 mm x 150 mm, 5 m) was used. The UV detector’s wavelength was 254 nm, and the mobile phase contained methanol at a concentration of 55% in 0.1% acetic acid [134]. The separations ran for about 5 min overall at a flow rate of 1 mL/min. The retention times were compared with pure standards to identify the peaks (Sigma-Aldrich, Deisenhofer-Germany). GAs and ethylene concentrations were determined using the appropriate integra standards.

### DNA isolation and ISSR profiling

Using Vejlupkova and Fowler [135] DNA extraction buffer, genomic DNA was extracted from *M. parviflora* leaves. Total extracted DNA was purified by column using the DNA Purification MiniSpin Kit (VIOGENE catalogue # PF1001) according to the manufacturer’s instructions. To verify DNA integrity, the isolated DNA was resolved on a 1% agarose gel electrophoresed in a 1X Tris-acetate-ethylenediaminetetraacetic acid (TAE) solution containing 0.5 µg/mL ethidium bromide. Using a UV-transilluminator, the gel stained with ethidium bromide was examined.

The polymerase chain reaction (PCR) was carried out in a Biometra thermal cycler using primers listed in Table (5) in a 25µL reaction volume. The PCR reaction and cycling were carried out according to Soliman et al. [100]. The PCR reaction was carried out twice for each primer to verify repeatability. The PCR reaction was carried out twice for each primer to verify repeatability. According to the manufacturer’s instructions, the reaction was carried out using *MyTaq* DNA polymerase (BIOLINE cat # BIO-21,108), in which 10 µM of each corresponding primer was included. The first five min of the PCR profile were passed at 95 °C, followed by 37 cycles of denaturation at 95 °C for one min, annealing at 50 °C for 30 s, and extension at 72 °C for two min. A final extension at 72 °C for 10 min was included. A 1.2% (w/v) agarose gel with a 1× TAE buffer containing 0.5 µg/mL ethidium bromide was used to separate the PCR products. With the use of a UV-transilluminator (made by Vilber Lourmat in Germany), the resolved bands were seen, and photographs were taken with a Nikon COOLPIX L820 digital camera. Only reproducible primers are taken into account for analysis. The ISSR markers were analysed using Quantity One Software 4.6.2.70. Binary data was used to score the band’s existence, with 1 denoting presence and 0 for absence. For each primer and treatment, the total numbers of unique, polymorphic and monomorphic bands as well as the percentage of polymorphism were calculated. Thermo Scientific #SM0241’s GeneRuler 100 bp DNA ladder was used to measure the size of the ISSR fragments.

The formula below was used to determine the percentage of genomic template stability (% of GTS): $=\left(1-\frac{a}{n}\right)X 100$ where n is the total number of bands in control samples, and a is the average number of changes in each DNA profile for each treatment [9, 136].

### Statistical analysis

The one- and two-way analysis of variance (ANOVA) procedures were performed to analyze the data using SPSS. By using a one-way ANOVA with a significance level of *p* < 0.05, the results were reported as the mean of five replicates plus standard error (SE). Using SPSS software version 15 (SPSS, Richmond, VA, USA) and a post hoc technique, the confidence interval was 95%. The effects of biopriming, drought, and their interactions on a number of growth, physiological, biochemical, and stress markers were also examined using a two-way ANOVA. Pearson’s correlation matrix between some measured parameters in *M. parviflora* plants was performed.

### Electronic supplementary material

Below is the link to the electronic supplementary material.

Supplementary Material 1

## Data Availability

Data Availability Statement: The relevant datasets supporting the results of this article are included within the article and the retrieved ITS sequence was deposited in NCBI GenBank under accession No. OP117371. https://www.ncbi.nlm.nih.gov/nuccore/OP117371.1.

## Declarations

### Experimental research and field studies on plants

All relevant institutional, national and international guidelines and legislation were compiled or adhered to in the production of this study.

## Abbreviations

AA: Ascorbic acid
ANOVA: Analysis of variance
CAT: Catalase
DW: Dry weight
FW: Fresh weight
GAE: Gallic acid equivalent
GTS: Genome template stability
IAA: Indoleacetic Acid
LOX: Lipoxygenase
MDA: Malondialdehyde
PAL: Phenylalanine ammonia-lyase
PCR: Polymerase chain reaction
POX: Peroxidase
QE: Quercetin equivalent
RWC: Relative water content
SE: Standard error
TAC: Total antioxidant capacity
TBA: Thiobarbituric acid
TFC: Total flavonoids content
TPC: Total phenolic content
TW: Turgid weight
WC: Water content
WHC: Water holding capacity
WSD: Water saturation deficient
DPPH: 2,2-diphenyl-1-picrylhydrazyl
HPLC: High-performance liquid chromatography
PGPR: Plant growth promoting rhizobacteria
GAs: Gibberellins
JA: Jasmonates
