# Supplementary material for: Enhancing drought tolerance in Malva parviflora plants through metabolic and genetic modulation using Beauveria bassiana inoculation
Source: BMC Plant Biol. 2024 Jul 11;24:662. doi: 10.1186/s12870-024-05340-w (PMC11238386; doi:10.1186/s12870-024-05340-w)
Supplement: Supplementary file 1 — Supplementary Material 1 [file 12870_2024_5340_MOESM1_ESM.docx]

Supplementary file for: " Enhancing drought tolerance in *Malva parviflora* plants through metabolic and genetic modulation using *Beauveria bassiana* inoculation"


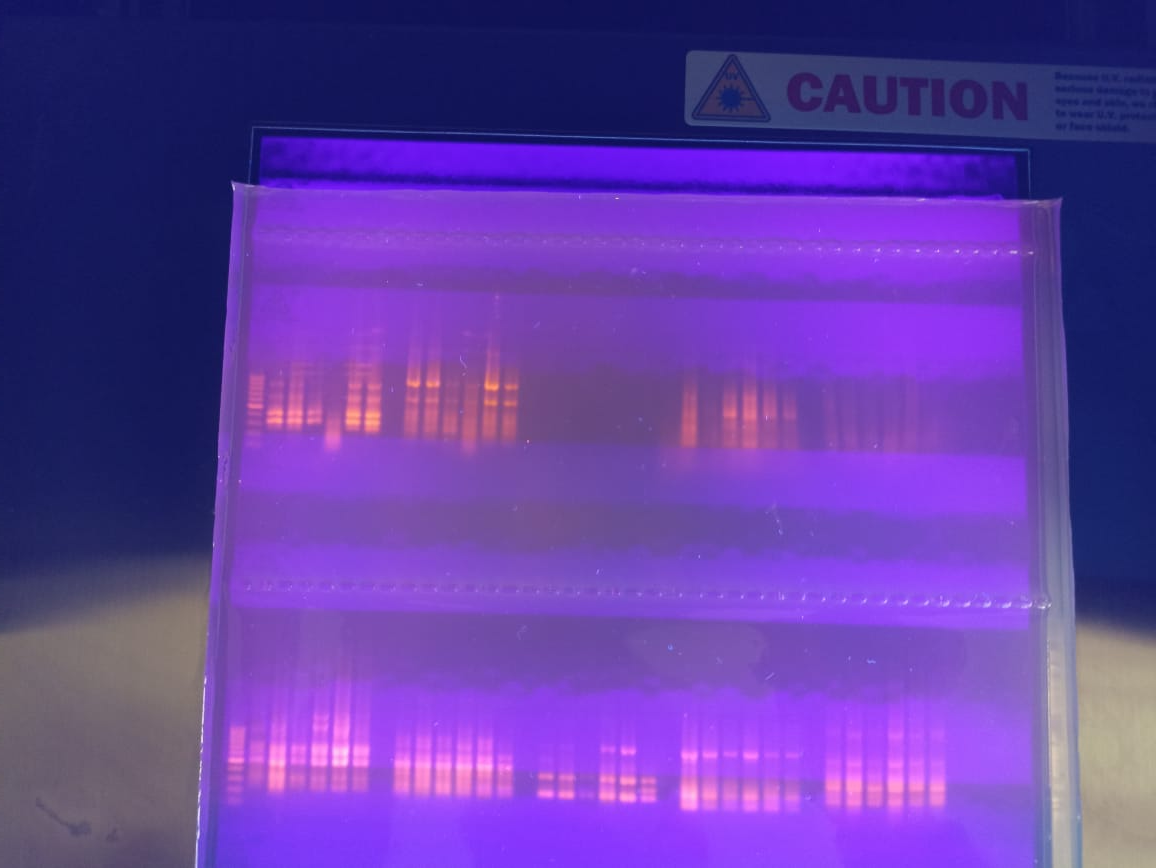


Primer ID: ISSR5 HB14 I-868 I-812 I-827

Primer ID: I-842 I-844 I-885 I-889 I891

**Supp 1:** Full-length image for Agarose gel electrophoresis for PCR products of ISSR fingerprints amplified in from *M. parviflora* plants DNA in response to different treatments by ten different primers: (Lower panel of the gel is Supplementary for figure 11a.


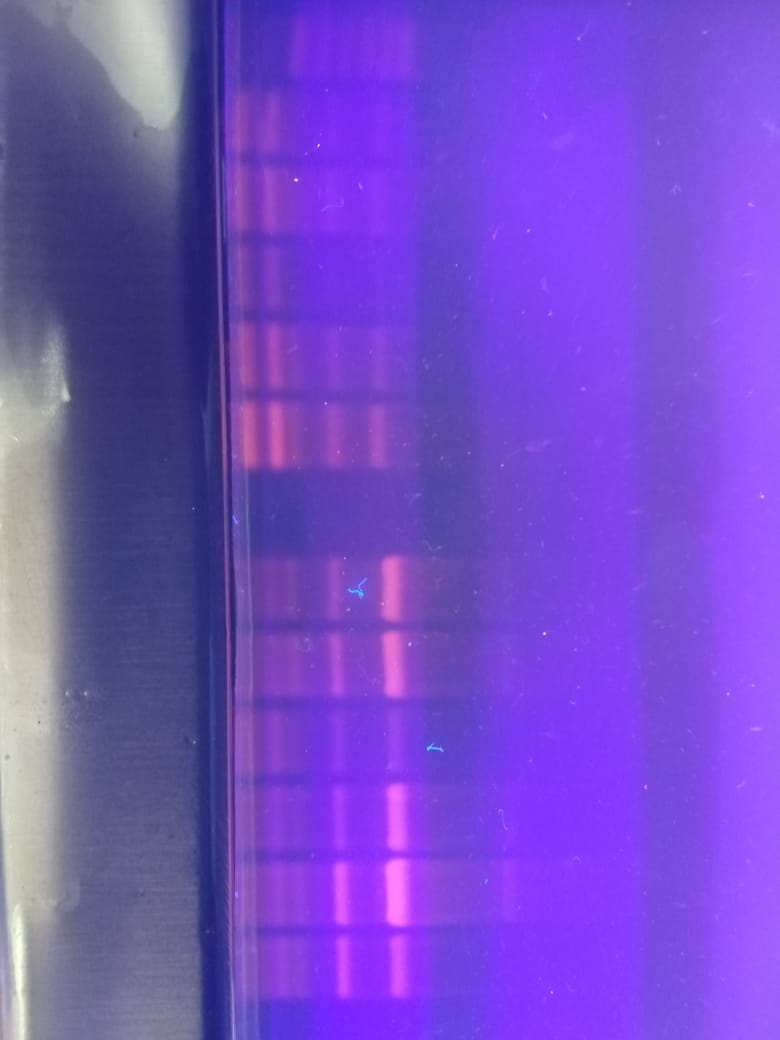


Primer ID ISSR5 HB14 HB14

**Supp 2:** Full-length image for Agarose gel electrophoresis for PCR products of ISSR fingerprints amplified in from *M. parviflora* plants DNA in response to different treatments: image is Supplementary for figure 11b.

|  |  |
| --- | --- |
| 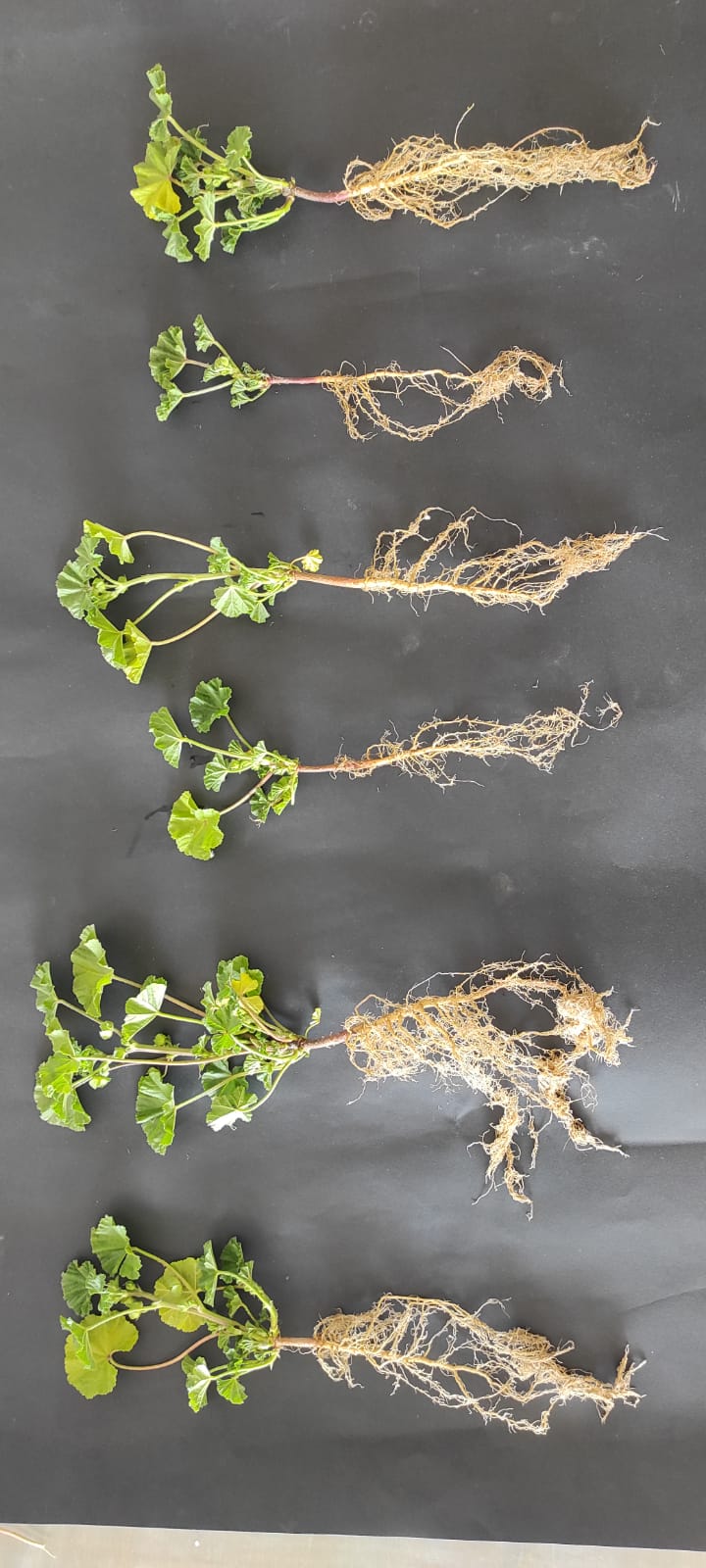 | |

**Supp 3:** *Malva parviflora* plants during the experiment and at the time of sampling


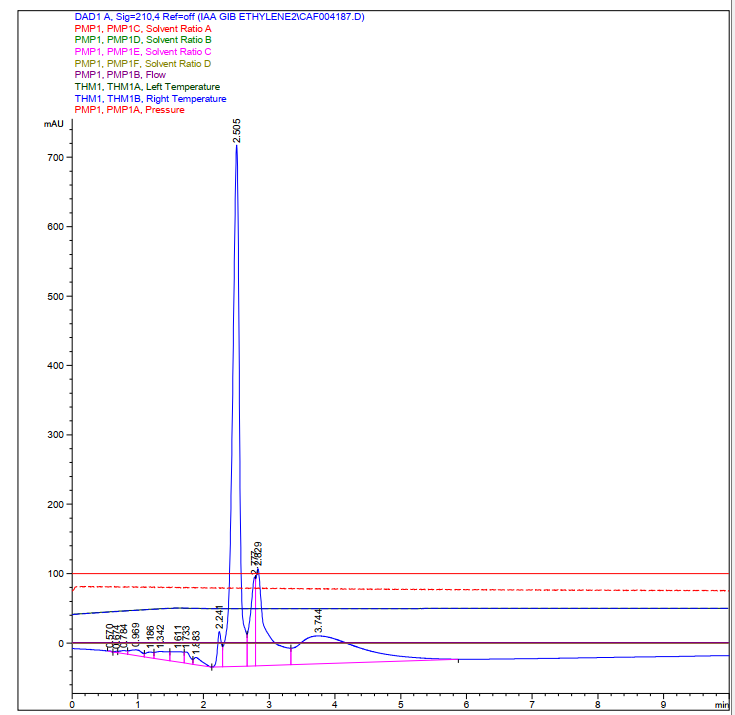


**Supp 4:** Chromatogram for standard gibberellins, ethylene and indol acetic acid.

**I-889**

**I-891**
